# Supplementary material for: Number of people treated for hepatitis C virus infection in 2014-2023 and applicable lessons for new HBV and HDV therapies
Source: J Hepatol. 2025 Aug;83(2):329–47. doi: 10.1016/j.jhep.2025.01.013 (PMC12278943; doi:10.1016/j.jhep.2025.01.013)
Supplement: Multimedia component 1 [file mmc1.pdf]

JHEP

CTAT methods table

Tables for a "Complete, Transparent, Accurate and Timely account" (CTAT) are now mandatory for all revised submissions. The aim is to enhance the reproducibility of methods.

- Only include the parts relevant to your study
- Refer to the CTAT in the main text as 'Supplementary CTAT Table'
- Do not add subheadings
- Add as many rows as needed to include all information
- Only include one item per row

If the CTAT form is not relevant to your study, please outline the reasons why:

Register-based case-control study.

1.1 Antibodies

Name	Citation	Supplier	Cat no.	Clone no.

1.2 Cell lines

Name	Citation	Supplier	Cat no.	Passage no.	Authentication test method

1.3 Organisms

Name	Citation	Supplier	Strain	Sex	Age	Overall n number

1.4 Sequence based reagents

Name	Sequence	Supplier

1.5 Biological samples

Description	Source	Identifier

1.6 Deposited data

Name of repository	Identifier	Link

1.7 Software

Software name	Manufacturer	Version
Crystal Ball	Oracle	11.1.3708.0

1.8 Other (*e.g.* drugs, proteins, vectors etc.)

1.9 Please provide the details of the corresponding methods author for the manuscript:

Homie Razavi, hrazavi@cdafound.org, +1-314-8054884

2.0 Please confirm for randomised controlled trials all versions of the clinical protocol are included in the submission. These will be published online as supplementary information.
