# Supplementary material for: Number of people treated for hepatitis C virus infection in 2014-2023 and applicable lessons for new HBV and HDV therapies
Source: J Hepatol. 2025 Aug;83(2):329–47. doi: 10.1016/j.jhep.2025.01.013 (PMC12278943; doi:10.1016/j.jhep.2025.01.013)
Supplement: Multimedia component 3 [file mmc3.pdf]

JOURNAL OF HEPATOLOGY

Number of people treated for hepatitis C virus infection in 2014-2023 and applicable lessons for new HBV and HDV therapies

The Polaris Observatory Collaborators*,†

Journal of Hepatology **2025**. vol. 83 | 329–347

Check for updates

See Editorial, pages 283–285

Background & Aims: The year 2023 marked the 10-year anniversary of the launch of direct-acting antivirals (DAAs) for the treatment of hepatitis C virus (HCV). Monitoring HCV treatment trends by country, region, and globally is important to assess progress toward the World Health Organization's 2030 elimination targets. Additionally, historical patterns can help predict the uptake of future therapies for other liver diseases.

Methods: The number of people living with HCV (PLHCV) treated between 2014–2023 across 119 countries was estimated using national HCV registries, reported DAA sales data, pharmaceutical companies' reports, and estimates provided by national experts. For the countries with no available data, the average estimate of the corresponding Global Burden of Disease region was used.

Results: An estimated 13,816,000 (95% uncertainty intervals: 13,221,000–16,415,000) PLHCV were treated, of whom 12,748,000 (12,226,000–15,231,000) were treated with DAAs, of which 11,081,000 (10,542,000–13,338,000) were sofosbuvir-based DAA regimens. Country-level data accounted for 97% of these estimates. In high-income countries, there was a 41% drop in treatment from its peak, and reimbursement was a large predictor of treatment. In low- and middle-income countries, price played an important role in expanding treatment access through the public and private markets, and treatment continues to increase slowly after a sharp drop at the end of the Egyptian national program.

Conclusions: In the last 10 years, 21% of all HCV infections were treated with DAAs. Regional and temporal variations highlight the importance of active screening strategies. Without program enhancements, the number of treated PLHCV stalled in every country/region, which may not reflect a lower prevalence but may instead reflect the diminishing returns of existing strategies.

© 2025 The Author(s). Published by Elsevier B.V. on behalf of European Association for the Study of the Liver. This is an open access article under the CC BY license (http://creativecommons.org/licenses/by/4.0/).

Introduction

The year 2023 marked the 10-year anniversary of the launch of direct-acting antivirals (DAAs) for the treatment of hepatitis C virus (HCV), an infection that affects 50 million people globally and leads to 244 thousand deaths annually, primarily from cancer and cirrhosis.[1] Compared to their predecessors, DAAs are characterized by high sustained viral responses (>95%), minimal side effects, and short treatment durations (~8–12 weeks).

The 69th World Health Assembly endorsed the World Health Organization's (WHO's) Global Health Sector Strategy for Viral Hepatitis with the goal of eliminating hepatitis infection as a public health threat by 2030.[2] This was followed by the WHO's global targets for management of HCV infections, including diagnosing and treating 90% and 80%, respectively, of all people living with HCV (PLHCV).[3] As such, estimates of treated PLHCV need to be tracked to determine how countries and regions are progressing toward achieving the elimination targets.[4,5]

Analysis of the trends in HCV treatment is also important since curative therapies for other viral infections (hepatitis B virus [HBV] and hepatitis delta virus [HDV]) are also in development. A study of HCV treatment patterns can help to prepare and forecast the uptake of these new therapies once approved.

The objectives of this study were to estimate the total number of DAA-treated PLHCV by country, region, and globally and to examine the treatment patterns across different regions.

* Corresponding author. Homie Razavi, 1120 W South Boulder Rd, Suite 102, Lafayette, CO 80026, USA. (T): 720-890-4848; (F): 720-890-3817.
E-mail address: hrazavi@cdafound.org.

† The full list of The Polaris Observatory Collaborators present in Appendix.

https://doi.org/10.1016/j.jhep.2025.01.013

Materials and methods

The number of treated PLHCV between 2014–2023 in each country was estimated using the following sources in descending order of priority: 1) national prescription drug/national registries, 2) reported DAA sales data, as provided by manufacturers under a confidentiality agreement and converted to treated PLHCV, 3) pharmaceutical companies' presentations to investors and analysts where their estimated number of treated PLHCV was reported, 4) pharmaceutical companies' local country office treatment estimates, 5) estimates provided by national experts working at major treatment centers via the annual Polaris Observatory update.

Priority was given to source 1 when it was clear that the public health system was accountable for treating PLHCV in the country (*e.g.*, Australia, Egypt, France, Georgia, Iceland, Italy, Norway, Spain, and the United Kingdom). For source 2, when sales data were reported by the number of pills, they were divided by 28 to convert to bottles. The number of bottles sold was then converted to treated PLHCV by dividing the number of bottles of sofosbuvir/velpatasvir, sofosbuvir/ledipasvir, elbasvir/grazoprevir, and ombitasvir/paritaprevir by 3 (12 weeks average duration of treatment) and of glecaprevir/pibrentasvir by 2 (8 weeks of treatment). For multi-pill combinations that also used daclatasvir, ravidasvir, ledipasvir, dasabuvir, ribavirin, and ritonavir, the unit sales of the nucleoside (*e.g.*, sofosbuvir, ombitasvir/paritaprevir) were used to estimate the number of treated PLHCV. Since sofosbuvir-based treatment accounted for most of all HCV treatments, the proportion of PLHCV on sofosbuvir-based regimens was calculated as a percentage of PLHCV on DAAs. In countries with access to generic sofosbuvir and daclatasvir,[6] it was assumed that all PLHCV were treated with sofosbuvir-based regimens. If a country had not reported their number of treated PLHCV in 2023, it was assumed to be the same as 2022 with the same percentage of DAA- and sofosbuvir-based regimens.

Sofosbuvir/velpatasvir/voxilaprevir was used as a second-line treatment and was not included in this analysis to prevent double counting. Similarly, 24 weeks of sofosbuvir/daclatasvir was used for treatment failures but was not considered in this analysis. The use of sofosbuvir/pegylated interferon/ribavirin was small relative to DAA therapies over the 10-year period and was also ignored. Traditional medicines, such as herbal therapies, were not considered.

To estimate the global and regional number of treated PLHCV, the regional treatment rate based on available country data for the Global Burden of Disease (GBD) regions[7] was used for countries with missing data. The following countries were excluded from the regional estimates due to their high treatment rate: Australia, Denmark, Egypt, Finland, France, Georgia, Iceland, Japan, Malaysia, New Zealand, the Netherlands, Norway, Portugal, the Republic of Korea, Rwanda, Saudi Arabia, the Seychelles, Slovenia, Spain, Sweden, Taiwan, and the United Kingdom; since in given years, they treated ≥10% of their HCV-infected population, which would not be representative of the countries with missing data in the same region.

For the base case estimate, the data source from the highest priority data source was selected (*e.g.*, national registry, method 1, was selected over sales data, method 2, and method 2 was selected over method 3). The data from the different sources were used to develop uncertainty intervals (UIs). Uncertainty analysis was conducted using Monte Carlo simulation with Latin Hypercube (a sampling size of 500) after running 10,000 trials in Crystal Ball® (Release 11.1.3708.0), an Excel® add-in by Oracle®. A binomial distribution was used for low (25% probability), base (50% probability), and high (25% probability) treatment estimates, with the 95% UI selected as the output. Given the asymmetry of the low, base, and high treated PLHCV estimates, UIs were deemed more appropriate than confidence intervals.

Results

An estimated 13,816,000 (95% UI: 13,221,000–16,415,000) PLHCV were treated in the last 10 years, of whom 12,748,000 (12,226,000–15,231,000) were treated with DAAs, including 11,081,000 (10,542,000–13,338,000) with sofosbuvir-based DAA regimens (Fig. 1A). In the last 10 years, 21% of the starting 62 million[8] HCV infections were treated with DAAs. Most of these estimates (97%) came from country-level data (Table 1), with 3% of the regional and global estimates from extrapolation of GBD regional treatment rate data. Country-reported treated patients accounted for 63% of the total estimates, followed by analysis of drug sales data (19%), company-reported estimates (14%), and expert inputs (4%).

The annual number of treated PLHCV is shown in Fig. 1B, demonstrating a rapid switch to DAAs in 2015, when they were approved widely, and a peak in 2019 due to the expansion of the Egyptian HCV elimination program in that year. The cumulative number of DAA treatments is shown in Fig. 1C, which demonstrates that by 2023, 87% were on a sofosbuvir-based regimen.

The annual regional and global estimates of DAA treatment are shown in Table 2, indicating the high treatment in WHO's Eastern Mediterranean region, lower middle-income group, and Asia. All the above came about due to the programs in Egypt and Pakistan (Fig. 2). Fig. 3 shows the annual number of treated PLHCV by World Bank income groups, demonstrating the treatment trends. This figure excludes the annual number of treated HCV infections in Egypt. In high-income countries (HICs), the number of treatments peaked in 2016, but it had already declined by 8% before the COVID-19 pandemic in 2020 resulted in a further 29% decline (Table 1). Similarly, in lower middle-income countries (excluding Egypt), treatment peaked in 2016 and declined by 22% before the COVID-19 pandemic resulted in an additional 44% decline in 2020. On the other hand, COVID-19's impact in the other regions was less pronounced. In 2020, treatment increased by 16% in upper middle-income countries and 650% in low-income countries. Finally, Fig. 2 shows the countries that accounted for 85% of all DAA treatments between 2014–2023. Egypt, Pakistan, the United States, and the European Union (EU) accounted for 67% of all HCV treatments. The EU was added to this graph due to its high treatment rate as a region, but the numbers reported for the EU overlap those of the EU member countries shown on the same graph.

Discussion

This study is unique as it presents the treatment pattern for the highly curative HCV therapies by country and region, and the described methodology can be used to monitor progress in

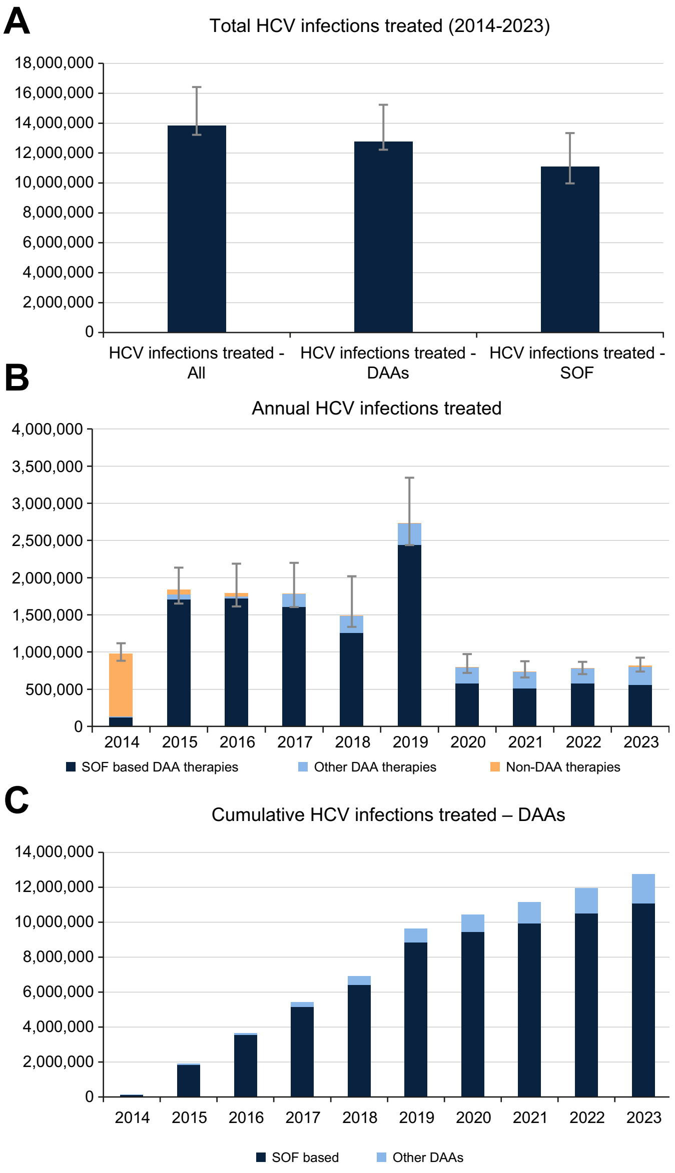

Fig. 1. Number of HCV infections treated globally in 2014-2023. (A) Total treated PLHCV between 2014-2023. (B) Annual number of treated PLHCV between 2014-2023. (C) Cumulative number of PLHCV treated with SOF-based regimens broken out. SOF-based = sofosbuvir-containing regimens. Other DAAs = elbasvir/grazoprevir, ombitasvir/paritaprevir, and glecaprevir/pibrentasvir-based therapies. Non-DAA therapies include interferon and protease-based therapies. DAAs, direct-acting antivirals; HCV, hepatitis C virus; PLHCV, people living with hepatitis C virus; SOF, sofosbuvir.

access to treatment. The lessons from this study may be applicable to new therapies being developed for other liver diseases.

Treated PLHCV

At the global level, the annual number of treated PLHCV peaked in 2019 (Table 2) when the Egyptian program was expanded, but the number of treatments did rebound in 2022 after the end of the Egyptian program and recovery from the COVID-19 pandemic.

At the WHO regional level, the increase in 2020 in the African Regional Office (AFRO) was mainly due to the expansion of Rwanda's national program (Tables 1 and 2). Rwanda was also responsible for the growth in treatment shown in low-income countries. The Eastern Mediterranean region's treated PLHCV estimate was dominated by the Egyptian and Pakistan programs, and the region accounted for 46% of global treated PLHCV between 2014-2023. The European region's treatment estimates peaked in 2019 coinciding with the expanded treatment in Uzbekistan, Ukraine, and Russia, while the EU's treatment estimates peaked in 2017, when treatment restrictions were removed. The EU accounted for 55% of the treated PLHCV in the European region. The Pan American Health Organization (PAHO) showed a peak in treatment in 2016 as a result of high treatment in the United States, as well as an expansion of treatment in Brazil. India was responsible for a treatment peak in 2016 in the Southeast Asian region. The Western Pacific region's treatment peaked in 2015 with the launch of Japan's program as well as an increase in DAA treatment in China. The WPRO region saw large fluctuations in treatment with the launch of national programs in Australia, Cambodia (Médecins Sans Frontières/UNITAID), Malaysia, Mongolia, New Zealand, the Philippines, and Vietnam.

At the national level, a discussion of global treatment of PLHCV starts with Egypt, as it alone accounted for 27% of all those treated in the studied period. As shown in Table 1, the country started its DAA treatment program in October 2014, but the annual number of treated PLHCV dropped by 2018 as the pool of those diagnosed, motivated, and warehoused was successfully treated. In 2018, the country initiated a national screening and treatment program, which led to 1.6 million Egyptians being treated in 2019 alone. The high uncertainty shown in Fig. 2 was due to the higher proportion of private-market HCV treatment before the national program kicked off.

The Egyptian national program[9] exemplifies the importance of: 1) a country's commitment to elimination – which led to removal of obstacles as they emerged (*e.g.*, initiating national screening as the number of treated cases declined), 2) adequately financing the national program, 3) implementing awareness and linkage to care programs, 4) negotiating with pharmaceutical companies to get access to the latest therapies as soon as they were launched in HICs, 5) collaborating with licensed and local drug manufacturers to ensure a steady supply of medicine at a low cost, 6) negotiating with diagnostic companies to get a steady supply of lab reagents, 7) training enough healthcare and community workers to support a national rollout, 8) creating a robust health information system to track progress, and 9) decentralizing service delivery to enable screening and linkage to care in rural villages and small towns. The Egyptian program showed that a passive HCV elimination program that focuses on treatment only will result in declining treatment numbers, and a full elimination program must be coupled with active screening, awareness, and linkage to care programs to achieve the WHO elimination goals.

Table 1. HCV infections treated with DAAs by country and region.

Country	Source	2014	2015	2016	2017	2018	2019	2020	2021	2022	2023	2014-2023
Afghanistan	DS			12 (11–15)	12 (11–15)	20 (18–30)	20 (18–30)	30 (30–40)	80 (70–100)	460 (410–580)	460 (410–580)	1,100 (980–1,400)
Algeria	DS		1,400 (1,300–2,000)	2,900 (2,600–4,200)				70 (60–100)				4,400 (3,900–6,400)
Argentina	20			2,200 (2,000–3,100)	2,200 (2,000–3,100)	2,300 (2,100–3,200)	2,160 (2,000–3,000)	1,900 (1,800–2,600)	1,400 (1,300–1,900)	1,700 (1,600–2,400)	1,700 (1,600–2,400)	15,600 (14,500–21,600)
Armenia	NCID				2,000 (2,000–3,400)	1,000 (1,000–1,700)	1,100 (1,100–1,800)	1,200 (1,200–2,000)	1,700 (1,700–2,800)	1,100 (1,100–1,800)	100 (100–170)	8,200 (8,200–13,700)
Australia	21–23	901 (810–1,200)	3,439 (3,100–4,400)	33,200 (29,900–42,500)	21,000 (18,900–26,900)	15,200 (13,700–19,500)	11,300 (10,200–14,500)	8,200 (7,400–10,500)	6,600 (5,900–8,500)	5,200 (4,700–6,700)	5,500 (5,000–7,000)	111,000 (99,500–142,000)
Austria	DS		2,000 (1,800–2,800)	2,000 (1,800–2,800)	2,000 (1,800–2,800)	1,900 (1,700–2,700)	1,800 (1,600–2,500)	1,200 (1,100–1,700)	1,100 (990–1,500)			12,000 (10,800–16,700)
Azerbaijan	DS			13 (10–30)		1 (1–3)	1,600 (1,300–4,000)		4 (3–10)	690 (550–1,700)	690 (550–1,700)	3,000 (2,400–7,500)
Bahrain	EC		50 (50–80)	150 (140–230)	100 (90–150)	100 (90–150)	100 (90–150)	100 (90–150)				600 (540–900)
Bangladesh	DS			110 (90–280)	2,000 (1,600–5,000)	5 (4–13)	100 (80–250)	200 (160–500)				2,400 (1,900–6,000)
Belarus	DS			30 (30–50)	150 (140–240)	1,500 (1,400–2,400)	4,600 (4,100–7,300)	1,600 (1,400–2,500)		6 (5–10)	6 (5–10)	7,900 (7,100–12,500)
Belgium	24		1,300 (1,200–1,700)	810 (730–1,000)	1,800 (1,600–2,300)	990 (890–1,300)	2,500 (2,300–3,200)	1,700 (1,500–2,200)	950 (860–1,200)	810 (730–1,000)	810 (730–1,000)	11,700 (10,500–15,000)
Bénin	MoH					16 (15–20)	140 (130–190)	80 (70–110)	40 (40–50)	160 (150–220)	60 (60–80)	500 (460–680)
Bolivia	DS						40 (40–60)	270 (240–390)		70 (60–100)	70 (60–100)	450 (410–650)
Bosnia and Herzegovina	DS				55 (50–70)	70 (60–90)	90 (80–110)	70 (60–90)	110 (100–140)			400 (360–490)
Brazil	25		7,459 (6,700–11,100)	41,260 (37,100–61,600)	15,200 (13,700–22,700)	12,200 (11,000–18,200)	36,700 (33,000–54,800)	19,200 (17,300–28,700)	14,900 (13,400–22,300)	16,200 (14,600–24,200)	16,400 (14,800–24,500)	180,000 (162,000–268,000)
Bulgaria	26,27		179 (160–220)	698 (630–870)	1,400 (1,300–1,800)	1,200 (1,100–1,500)	930 (840–1,200)	810 (730–1,000)	810 (730–1,000)			6,000 (5,400–7,500)
Burkina Faso	DS			10 (9–13)		830 (750–1,100)	830 (750–1,100)	2,600 (2,300–3,400)	3,300 (3,000–4,300)	2,800 (2,500–3,600)	2,800 (2,500–3,600)	13,200 (11,900–17,100)

(*continued on next page*)

Table 1. (*continued*)

Country	Source	2014	2015	2016	2017	2018	2019	2020	2021	2022	2023	2014-2023
Burundi	DS			630 (570–990)	1,200 (1,100–1,900)	270 (240–420)	280 (250–440)	360 (320–570)	470 (420–740)	80 (70–130)	80 (70–130)	3,400 (3,000–5,300)
Cambodia	NDB,[28,29]		50 (50–60)	310 (280–400)	3,000 (2,700–3,900)	8,600 (7,700–11,100)	5,400 (4,900–7,000)	2,800 (2,500–3,600)	2,000 (1,800–2,600)	280 (250–360)	400 (360–520)	22,800 (20,600–29,400)
Cameroon	DS			1,200 (1,100–2,300)	1,900 (1,700–3,600)	4,000 (3,600–7,600)		810 (730–1,500)	540 (490–1,000)	540 (490–1,000)	540 (490–1,000)	9,500 (8,600–18,200)
Canada	[30], EC		11,100 (10,000–14,100)	10,500 (9,500–13,400)	14,900 (13,400–19,000)	17,900 (16,100–22,800)	16,300 (14,700–20,800)	11,300 (10,200–14,400)	11,000 (9,900–14,000)	11,000 (9,900–14,000)	11,000 (9,900–14,000)	115,000 (104,000–146,000)
Chad	DS							1 (1–3)		310 (250–780)	310 (250–780)	620 (500–1,600)
Chile	DS		120 (100–300)	60 (50–150)	60 (50–150)	260 (210–650)	220 (180–550)		1 (1–3)			720 (580–1,800)
China Mainland	DS		70,000 (66,400–212,000)		200 (190–610)	390 (370–1,200)	1,000 (950–3,000)	52,100 (49,400–158,000)	93,000 (88,200–281,000)	94,700 (89,800–287,000)	94,700 (89,800–287,000)	406,000 (385,000–1,229,000)
Colombia	31			550 (500–750)	560 (500–770)	1,100 (990–1,500)	580 (520–790)	490 (440–670)	670 (600–920)	890 (800–1,200)	1,200 (1,100–1,600)	6,000 (5,400–8,300)
Congo, Republic of the	DS			30 (30–40)		70 (60–90)	50 (50–60)	13 (12–16)	60 (50–80)	2 (2–3)	2 (2–3)	230 (200–280)
Côte d'Ivoire	DS			50 (40–130)		5,000 (4,000–12,500)						5,100 (4,000–12,600)
Croatia	EC		99 (90–130)	179 (160–230)	340 (310–430)	440 (400–560)	474 (430–600)	360 (320–460)	340 (310–430)	250 (230–320)	300 (270–380)	2,800 (2,500–3,500)
Cuba	EC			15 (14–20)		5 (5–7)	80 (70–110)					100 (90–130)
Czechia	[27], EC		257 (230–320)	570 (520–720)	620 (560–780)	650 (590–820)	1,400 (1,300–1,800)	2,300 (2,100–2,900)	1,700 (1,500–2,100)	2,600 (2,400–3,300)	2,600 (2,400–3,300)	12,700 (11,500–16,000)
Denmark	INFcare		630 (570–790)	510 (460–640)	450 (410–560)	750 (680–940)	2,000 (1,800–2,500)	800 (720–1,000)	500 (450–630)	500 (450–630)	500 (450–630)	6,600 (6,000–8,300)
Dominican Republic	EC		41 (30–100)	300 (240–750)	800 (640–2,000)	1,200 (960–3,000)	1,800 (1,400–4,500)					4,100 (3,300–10,400)
Egypt	9		827,000 (744,000–1,087,000)	342,000 (308,000–450,000)	479,000 (431,000–630,000)	226,000 (203,000–297,000)	1,600,000 (1,440,000–2,103,000)	15,000 (13,500–19,700)	5,000 (4,500–6,600)	3,000 (2,700–3,900)	310 (280–410)	3,497,000 (3,148,000–4,597,000)
Eritrea	DS						100 (90–130)			270 (240–340)	270 (240–340)	640 (580–800)

(*continued on next page*)

Table 1. (*continued*)

Country	Source	2014	2015	2016	2017	2018	2019	2020	2021	2022	2023	2014-2023
Estonia	32		450 (410–650)	604 (540–870)	562 (510–810)	950 (860–1,400)	980 (880–1,400)	630 (570–910)	1,200 (1,100–1,700)		780 (700–1,100)	6,200 (5,500–8,900)
Ethiopia	DS		2,000 (1,800–2,800)	850 (770–1,200)		320 (290–450)		620 (560–880)	890 (810–1,300)	1,200 (1,100–1,700)	280 (250–400)	6,200 (5,600–8,700)
Finland	EC		75 (70–100)	160 (140–210)	300 (270–380)	1,368 (1,200–1,800)	1,970 (1,800–2,500)	2,000 (1,800–2,600)	2,100 (1,900–2,700)	2,000 (1,800–2,600)	1,700 (1,500–2,200)	11,700 (10,500–15,000)
France	INVS		19,400 (17,500–24,900)	16,000 (14,400–20,500)	19,600 (17,600–25,100)	14,000 (12,600–18,000)	11,400 (10,300–14,600)	7,100 (6,400–9,100)	5,900 (5,300–7,600)	5,500 (5,000–7,100)	5,500 (5,000–7,100)	104,000 (94,000–134,000)
Georgia	Georgia CDC		6,000 (5,400–7,500)	21,600 (19,400–27,100)	15,000 (13,500–18,800)	10,300 (9,300–12,900)	12,400 (11,200–15,600)	8,500 (7,700–10,700)	4,000 (3,600–5,000)	4,400 (4,000–5,500)	4,400 (4,000–5,500)	86,600 (77,900–109,000)
Germany	DS		21,500 (19,400–27,000)	14,200 (12,800–17,800)	12,800 (11,500–16,100)	10,600 (9,500–13,300)	9,700 (8,700–12,200)	7,600 (6,800–9,500)	5,600 (5,000–7,000)	6,000 (5,400–7,500)	7,900 (7,100–9,900)	95,900 (86,300–121,000)
Ghana	DS			4 (3–10)	1,300 (1,000–3,300)		17 (14–40)	17 (14–40)	30 (20–80)	310 (250–780)	310 (250–780)	2,000 (1,600–5,000)
Greece	EC		1,500 (1,400–2,100)	1,500 (1,400–2,100)	1,900 (1,700–2,600)	1,600 (1,400–2,200)	1,200 (1,100–1,600)	1,300 (1,200–1,800)	1,200 (1,100–1,600)	1,100 (990–1,500)	1,600 (1,400–2,200)	12,900 (11,600–17,700)
Guatemala	DS			10 (9–13)	40 (40–50)				1,800 (1,600–2,300)			1,900 (1,700–2,300)
Guinea	DS					2 (2–5)		3 (2–8)		270 (220–680)	270 (220–680)	550 (440–1,400)
Guyana	DS				1 (1–3)				1 (1–3)	1,700 (1,400–4,300)	1,700 (1,400–4,300)	3,400 (2,700–8,500)
Haiti	DS					3 (2–8)		90 (70–230)	16 (13–40)			110 (90–270)
Hong Kong	EC		85 (80–130)	124 (110–190)	16 (14–20)	40 (40–60)	7 (6–11)	390 (350–590)				660 (600–1,000)
Hungary	27,33			931 (840–1,200)	923 (830–1,200)	2,479 (2,200–3,100)	1,400 (1,300–1,800)	900 (810–1,100)	500 (450–630)	750 (680–950)	650 (590–820)	8,500 (7,700–10,800)
Iceland	Trap HepC		30 (30–40)	480 (430–620)	170 (150–220)	100 (90–130)	80 (70–100)	80 (70–100)	50 (50–60)	70 (60–90)	70 (60–90)	1,100 (1,000–1,500)
India	DS		60,200 (54,200–98,100)	194,000 (175,000–316,000)	182,000 (164,000–297,000)	180,000 (162,000–293,000)	90,000 (81,000–147,000)					706,000 (636,000–1,151,000)
Indonesia	34		1,300 (1,200–4,400)	6,600 (5,900–22,100)	1,400 (1,300–4,700)	2,000 (1,800–6,700)	2,200 (2,000–7,400)	310 (280–1,000)	1,600 (1,400–5,400)	1,600 (1,400–5,400)	1,600 (1,400–5,400)	18,600 (16,700–62,300)

(*continued on next page*)

Table 1. (*continued*)

Country	Source	2014	2015	2016	2017	2018	2019	2020	2021	2022	2023	2014-2023
Iran	EC		5,300 (5,100–8,600)	2,545 (2,400–4,100)	5,949 (5,700–9,700)	4,856 (4,700–7,900)			2,400 (2,300–3,900)			21,000 (20,200–34,200)
Iraq	DS		240 (220–850)	2,000 (1,800–7,100)	60 (50–210)	40 (40–140)	100 (90–350)	60 (50–210)	20 (18–70)	20 (18–70)	20 (18–70)	2,600 (2,300–9,000)
Ireland	HSE		350 (320–470)	540 (490–720)	1,100 (990–1,500)	1,600 (1,400–2,100)	1,200 (1,100–1,600)	530 (480–710)	530 (480–710)	530 (480–710)	570 (510–760)	7,000 (6,300–9,300)
Israel	EC		1,500 (1,400–1,900)	1,800 (1,700–2,300)	1,500 (1,400–1,900)	3,400 (3,100–4,300)	2,400 (2,200–3,000)	1,700 (1,600–2,100)	1,200 (1,100–1,500)	1,000 (930–1,300)	1,000 (930–1,300)	15,500 (14,300–19,400)
Italy	35		31,200 (28,100–46,800)	34,400 (31,000–51,600)	44,400 (40,000–66,600)	56,300 (50,700–84,400)	36,300 (32,700–54,400)	15,400 (13,900–23,100)	14,500 (13,100–21,700)	13,100 (11,800–19,600)	13,100 (11,800–19,600)	259,000 (233,000–388,000)
Japan	MHLW & NDB		92,200 (83,000–163,000)	63,400 (57,100–112,000)	34,900 (31,400–61,900)	36,800 (33,100–65,200)	20,400 (18,400–36,200)	15,100 (13,600–26,800)	12,400 (11,200–22,000)	9,200 (8,300–16,300)	9,200 (8,300–16,300)	294,000 (264,000–520,000)
Jordan	DS			400 (360–580)	500 (450–720)	420 (380–610)						1,300 (1,200–1,900)
Kazakhstan	rcez.kz		450 (410–1,200)	450 (410–1,200)	1,900 (1,700–5,100)	2,000 (1,800–5,300)	15,400 (13,900–41,100)	7,700 (6,900–20,600)	6,600 (5,900–17,600)	6,800 (6,100–18,200)	11,000 (9,900–29,400)	52,300 (47,100–140,000)
Kenya	DS		40 (40–120)	570 (510–1,700)	90 (80–280)	30 (30–90)	1,200 (1,100–3,700)	1,200 (1,100–3,700)	190 (170–580)	1,600 (1,400–4,900)	1,600 (1,400–4,900)	6,500 (5,900–20,000)
Korea, Republic of	KCDC		1,500 (1,400–1,900)	13,200 (11,900–16,900)	13,000 (11,700–16,700)	12,600 (11,300–16,200)	12,200 (11,000–15,700)	7,600 (6,800–9,800)	6,100 (5,500–7,800)	5,600 (5,000–7,200)	5,500 (5,000–7,100)	77,300 (69,600–99,300)
Kyrgyzstan	DS			1,100 (990–1,400)	1,000 (900–1,200)	970 (870–1,200)	1,800 (1,600–2,200)	2,300 (2,100–2,900)	450 (410–560)			7,600 (6,900–9,500)
Laos	DS			1 (1–3)	3 (2–8)	50 (40–130)	690 (570–1,700)	3,900 (3,200–9,800)	530 (440–1,300)	1 (1–3)	1 (1–3)	5,200 (4,300–12,900)
Latvia	27,36		910 (820–1,100)	410 (370–510)	1,260 (1,100–1,600)	1,700 (1,500–2,100)	2,500 (2,300–3,100)	2,600 (2,300–3,300)	1,500 (1,400–1,900)	1,100 (990–1,400)	1,100 (990–1,400)	13,100 (11,800–16,300)
Lebanon	DS			330 (330–650)	220 (220–430)			10 (10–20)	10 (10–20)	30 (30–60)	30 (30–60)	630 (630–1,200)
Liberia	DS							970 (870–1,200)				970 (870–1,200)
Libya	DS		1,700 (1,500–2,600)					970 (870–1,500)	830 (750–1,300)			3,500 (3,200–5,300)
Lithuania	27,37		423 (380–530)	332 (300–420)	766 (690–960)	1,176 (1,100–1,500)	1,900 (1,700–2,400)	930 (840–1,200)	950 (860–1,200)	1,600 (1,400–2,000)	3,200 (2,900–4,000)	11,300 (10,100–14,100)

(*continued on next page*)

Table 1. (*continued*)

Country	Source	2014	2015	2016	2017	2018	2019	2020	2021	2022	2023	2014-2023
Luxembourg	DS		150 (140–190)	300 (270–380)	200 (180–250)	130 (120–170)	180 (160–230)	120 (110–150)	130 (120–170)	150 (140–190)	130 (120–170)	1,500 (1,300–1,900)
Malaysia	[38], EC			330 (310–420)	330 (310–420)	1,200 (1,100–1,500)	3,200 (3,000–4,000)	3,504 (3,300–4,400)	4,200 (3,900–5,300)	4,800 (4,500–6,100)	4,800 (4,500–6,100)	22,400 (20,800–28,300)
Malta	DS		40 (30–100)	70 (60–180)	100 (80–250)			1 (1–3)				210 (170–530)
Mauritania	DS			220 (200–270)								220 (200–270)
Mauritius	DS			300 (240–750)	6 (5–15)	100 (80–250)	4 (3–10)					410 (330–1,000)
Mexico	MoH			2,400 (2,200–3,300)	1,035 (970–1,400)	2,700 (2,500–3,700)	4,320 (4,000–5,900)	5,500 (5,200–7,500)	5,500 (5,200–7,500)	15,000 (14,100–20,500)	7,900 (7,400–10,800)	44,400 (41,600–60,600)
Moldova	DS			4 (3–10)			3,200 (2,600–8,000)	400 (320–1,000)	130 (100–330)			3,700 (3,000–9,300)
Mongolia	NCCD		280 (250–390)	10,400 (9,400–14,400)	17,700 (15,900–24,500)	15,600 (14,000–21,600)	10,800 (9,700–15,000)	6,900 (6,200–9,600)	1,100 (990–1,500)	2,100 (1,900–2,900)	2,100 (1,900–2,900)	67,000 (60,300–92,700)
Morocco	MoH			1,600 (1,500–2,400)	1,600 (1,500–2,400)	1,300 (1,200–1,900)	1,200 (1,100–1,800)	1,000 (950–1,500)	1,000 (950–1,500)			7,700 (7,400–11,500)
Myanmar	DS		1,900 (1,700–4,200)	17,500 (15,800–38,400)	8,000 (7,200–17,600)	7,000 (6,300–15,400)	3,000 (2,700–6,600)	3,700 (3,300–8,100)	1,300 (1,200–2,900)	680 (610–1,500)	680 (610–1,500)	43,800 (39,400–96,000)
Nepal	DS		3,300 (3,000–4,100)	1,700 (1,500–2,100)	230 (210–290)	340 (310–430)	170 (150–210)	210 (190–260)	1,600 (1,400–2,000)			7,600 (6,800–9,400)
Netherlands	39	58 (50–70)	819 (740–1,000)	1,509 (1,400–1,900)	600 (540–760)	529 (480–670)	355 (320–450)	241 (220–310)	218 (200–280)	186 (170–240)	132 (120–170)	4,600 (4,200–5,900)
New Zealand	DS		534 (480–800)	1,412 (1,300–2,100)	1,486 (1,300–2,200)	840 (760–1,300)	3,500 (3,200–5,300)	1,000 (900–1,500)	810 (730–1,200)	710 (640–1,100)	710 (640–1,100)	11,000 (9,900–16,500)
Nicaragua	DS									50 (40–130)	50 (40–130)	100 (80–250)
Nigeria	DS		740 (670–930)	1,200 (1,100–1,500)	1,800 (1,600–2,300)	400 (360–500)	400 (360–500)	4,200 (3,800–5,300)	270 (240–340)			9,000 (8,100–11,300)
Norway	40	740 (670–950)	861 (770–1,100)	974 (880–1,200)	1,849 (1,700–2,400)	3,098 (2,800–4,000)	2,100 (1,900–2,700)	1,300 (1,200–1,700)	930 (840–1,200)	768 (690–980)	850 (770–1,100)	13,500 (12,100–17,300)
Oman	EC		70 (60–100)	410 (370–580)	500 (450–700)	500 (450–700)	500 (450–700)	200 (180–280)	200 (180–280)	500 (450–700)	300 (270–420)	3,200 (2,900–4,500)

(*continued on next page*)

Table 1. (*continued*)

Country	Source	2014	2015	2016	2017	2018	2019	2020	2021	2022	2023	2014-2023
Pakistan	41		228,000 (205,000–291,000)	360,000 (324,000–459,000)	349,000 (314,000–445,000)	354,000 (319,000–452,000)	324,000 (292,000–413,000)	154,000 (139,000–196,000)	143,000 (129,000–182,000)	176,000 (158,000–225,000)	194,000 (175,000–247,000)	2,282,000 (2,054,000–2,911,000)
Peru	DS				6 (5–15)	6 (5–15)	6 (5–15)	1 (1–3)	290 (230–730)			310 (250–770)
Philippines	DS		130 (120–170)	180 (160–230)	2,300 (2,100–2,900)	5,100 (4,600–6,500)	1,500 (1,400–1,900)	640 (580–820)	3,700 (3,300–4,700)	210 (190–270)	210 (190–270)	14,000 (12,600–17,800)
Poland	42		4,100 (3,700–5,300)	8,000 (7,200–10,400)	11,115 (10,000–14,400)	6,816 (6,100–8,900)	8,330 (7,500–10,800)	3,100 (2,800–4,000)	2,600 (2,300–3,400)	3,100 (2,800–4,000)	3,400 (3,100–4,400)	50,600 (45,500–65,700)
Portugal	Atlas ECDC		9,700 (8,700–12,100)	6,800 (6,100–8,500)	4,900 (4,400–6,100)	4,700 (4,200–5,900)	3,900 (3,500–4,900)	2,300 (2,100–2,900)	2,300 (2,100–2,900)	2,200 (2,000–2,800)	2,200 (2,000–2,800)	39,000 (35,100–48,800)
Qatar	DS		237 (210–310)	360 (320–470)	550 (500–720)	320 (290–420)	600 (540–790)	150 (140–200)	130 (120–170)	130 (120–170)	130 (120–170)	2,600 (2,300–3,400)
Romania	43		286 (260–380)	5,478 (4,900–7,300)	10,200 (9,200–13,600)	5,800 (5,200–7,700)	14,400 (13,000–19,100)	6,900 (6,200–9,200)	4,400 (4,000–5,800)	5,900 (5,300–7,800)	5,900 (5,300–7,800)	59,300 (53,300–78,700)
Russia	EC			4,540 (4,100–6,200)	7,901 (7,100–10,700)	7,129 (6,400–9,700)	12,066 (10,900–16,400)	22,230 (20,000–30,200)	22,892 (20,600–31,100)	23,463 (21,100–31,900)	30,400 (27,400–41,400)	131,000 (118,000–178,000)
Rwanda	44		800 (720–2,100)	1,000 (910–2,600)	3,300 (3,000–8,500)	6,600 (6,000–16,900)	800 (720–2,100)	16,900 (15,300–43,400)	17,400 (15,800–44,700)	23,100 (20,900–59,300)	2,200 (2,000–5,600)	72,100 (65,300–185,000)
Saudi Arabia	MoH			7,500 (7,000–10,300)	4,600 (4,300–6,300)	2,400 (2,200–3,300)	1,800 (1,700–2,500)	1,300 (1,200–1,800)	780 (730–1,100)	1,700 (1,600–2,300)	1,500 (1,400–2,100)	21,600 (20,200–29,700)
Seychelles	DS			70 (60–180)	70 (60–180)		2 (2–5)		130 (120–330)			270 (240–680)
Singapore	DS			90 (80–130)	550 (500–760)	30 (30–40)	1,400 (1,300–1,900)	940 (850–1,300)	90 (80–130)	9 (8–13)	9 (8–13)	3,100 (2,800–4,300)
Slovakia	27,45			405 (360–510)	350 (320–440)	396 (360–500)	400 (360–510)	230 (210–290)	230 (210–290)	270 (240–340)	360 (320–460)	2,600 (2,400–3,300)
Slovenia	EC		129 (120–180)	178 (160–250)	260 (230–370)	250 (230–360)	102 (90–150)	100 (90–140)	120 (110–170)		140 (130–200)	1,300 (1,200–1,800)
South Africa	EC, MoH			100 (90–150)	300 (270–440)	250 (230–370)	200 (180–290)	250 (230–370)	800 (720–1,200)	3,400 (3,100–5,000)	6,100 (5,500–9,000)	11,400 (10,300–16,800)
Spain	46		29,100 (26,200–39,300)	28,700 (25,800–38,800)	29,000 (26,100–39,200)	26,600 (23,900–36,000)	15,900 (14,300–21,500)	8,400 (7,600–11,400)	7,400 (6,700–10,000)	7,000 (6,300–9,500)	6,000 (5,400–8,100)	158,000 (142,000–214,000)
Sri Lanka	DS			12 (10–30)	60 (50–150)	70 (60–180)	50 (40–130)	2,300 (1,800–5,800)	130 (100–330)	280 (220–700)	280 (220–700)	3,200 (2,600–8,000)
Suriname	DS									80 (60–200)		80 (60–200)

(*continued on next page*)

Table 1. (*continued*)

Country	Source	2014	2015	2016	2017	2018	2019	2020	2021	2022	2023	2014-2023
Sweden	47,48	1,300 (1,200–1,600)	2,300 (2,100–2,900)	2,500 (2,300–3,100)	2,100 (1,900–2,600)	5,800 (5,200–7,300)	4,800 (4,300–6,000)	2,400 (2,200–3,000)	2,100 (1,900–2,600)	1,700 (1,500–2,100)	1,800 (1,600–2,300)	26,800 (24,100–33,500)
Switzerland	DS	528 (480–820)	2,300 (2,100–3,600)	1,900 (1,700–3,000)	3,000 (2,700–4,700)	3,200 (2,900–5,000)	1,800 (1,600–2,800)	980 (880–1,500)	880 (790–1,400)	710 (640–1,100)	790 (710–1,200)	16,100 (14,500–25,100)
Taiwan	EC		500 (450–680)	899 (810–1,200)	10,536 (9,500–14,300)	19,600 (17,600–26,700)	45,800 (41,200–62,300)	36,200 (32,600–49,200)	20,000 (18,000–27,200)	15,800 (14,200–21,500)	13,800 (12,400–18,800)	163,000 (147,000–222,000)
Tajikistan	DS			1,000 (900–1,300)	1,500 (1,400–2,000)	2,000 (1,800–2,700)	480 (430–640)	6,400 (5,800–8,600)	410 (370–550)	70 (60–90)	70 (60–90)	11,900 (10,700–16,000)
Tanzania	DS						1 (1–3)	2,100 (1,700–5,300)		1 (1–3)	1 (1–3)	2,100 (1,700–5,300)
Thailand	DS				3,000 (3,000–5,400)	2,200 (2,200–3,900)	3,800 (3,800–6,800)	2,700 (2,700–4,800)				11,700 (11,700–21,000)
Tunisia	DS					1,100 (990–1,400)	80 (70–100)	120 (110–150)	240 (220–300)			1,500 (1,400–1,900)
Türkiye	DS		4,900 (4,400–7,200)	5,600 (5,000–8,200)	5,300 (4,800–7,800)	5,300 (4,800–7,800)	5,500 (5,000–8,100)	5,600 (5,000–8,200)	5,600 (5,000–8,200)	2,700 (2,400–4,000)	3,100 (2,800–4,600)	43,600 (39,200–64,200)
Turkmenistan	DS		420 (380–530)	380 (340–480)	540 (490–680)	170 (150–210)	780 (700–980)	11,900 (10,700–14,900)	6,600 (5,900–8,300)	20 (18–30)	20 (18–30)	20,800 (18,700–26,000)
Uganda	DS		40 (30–100)	30 (20–80)	30 (20–80)	100 (80–250)	30 (20–80)	3,400 (2,700–8,500)	60 (50–150)	4 (3–10)	4 (3–10)	3,700 (3,000–9,200)
Ukraine	DS		550 (500–1,300)	2,400 (2,200–5,500)	749 (670–1,700)	1,156 (1,000–2,600)	8,640 (7,800–19,700)	8,600 (7,700–19,600)	16,300 (14,700–37,200)	12,800 (11,500–29,200)		51,200 (46,100–117,000)
United Kingdom	49–52		8,100 (7,300–10,800)	12,100 (10,900–16,200)	14,600 (13,100–19,500)	15,200 (13,700–20,300)	15,400 (13,900–20,600)	9,200 (8,300–12,300)	11,500 (10,400–15,400)	11,000 (9,900–14,700)	11,000 (9,900–14,700)	108,000 (97,300–144,000)
United States	DS	126,000 (113,000–158,000)	260,000 (234,000–325,000)	231,000 (208,000–289,000)	208,000 (187,000–260,000)	187,000 (168,000–234,000)	195,000 (176,000–244,000)	147,000 (132,000–184,000)	133,000 (120,000–166,000)	142,000 (128,000–178,000)	142,000 (128,000–178,000)	1,771,000 (1,594,000–2,214,000)
Uzbekistan	EC			15,000 (13,500–22,600)	9,300 (8,400–14,000)	8,500 (7,700–12,800)	29,100 (26,200–43,800)	9,900 (8,900–14,900)	11,500 (10,400–17,300)	14,500 (13,100–21,800)	36,200 (32,600–54,400)	134,000 (121,000–202,000)
Vietnam	DS		4,500 (4,100–10,000)	5,100 (4,600–11,300)	10,800 (9,700–24,000)	12,000 (10,800–26,600)	10,000 (9,000–22,200)	12,900 (11,600–28,600)	3,000 (2,700–6,700)	60 (50–130)	60 (50–130)	58,400 (52,600–130,000)
Zimbabwe	DS			60 (50–150)			19 (16–50)			5 (4–13)	5 (4–13)	90 (70–220)
Other	DS			169,000 (152,000–264,000)	132,000 (119,000–207,000)	102,000 (91,800–160,000)	59,300 (53,400–92,800)					462,000 (416,000–723,000)

DAAs, direct-acting antivirals; DS, drug sales data; EC, expert consensus; HCV, hepatitis C virus; HSE, National Hepatitis C Treatment Programme, Ireland; INVS, Institut de Veille Sanitaire; MHLW, Ministry of Health, Labor and Welfare; MoH, Ministry of Health; MSF, Médecins Sans Frontières; NCCD, National Center for Communicable Diseases; NCID, National Center for Infectious Diseases; NDB, National Database; PHAC, Public Health Agency Canada.

Table 2. HCV infections treated with DAAs by WHO regions, World Bank income group, the European Union, continent, and globally.

**WHO region**	**2014**	**2015**	**2016**	**2017**	**2018**	**2019**	**2020**	**2021**	**2022**	**2023**	**2014-2023**
African regional office (AFRO)		8,400 (7,800–9,400)	10,400 (9,800–11,700)	18,800 (17,900–21,900)	23,200 (22,000–26,500)	6,100 (6,000–32,800)	37,100 (35,900–84,800)	27,200 (25,100–52,400)	38,300 (33,400–45,100)	19,300 (18,300–42,000)	189,000 (182,000–318,000)
Eastern Mediterranean regional office (EMRO)		1,066,000 (856,000–1,329,000)	721,000 (582,000–895,000)	844,000 (773,000–1,050,000)	594,000 (535,000–961,000)	1,930,000 (1,549,000–2,411,000)	178,000 (175,000–242,000)	157,000 (128,000–230,000)	184,000 (149,000–230,000)	200,000 (162,000–253,000)	5,873,000 (4,913,000–7,602,000)
European regional office (EURO)	2,600 (2,200–3,100)	157,000 (155,000–192,000)	201,000 (190,000–254,000)	223,000 (216,000–273,000)	218,000 (216,000–306,000)	247,000 (241,000–294,000)	175,000 (172,000–223,000)	152,000 (144,000–166,000)	143,000 (138,000–157,000)	168,000 (159,000–187,000)	1,688,000 (1,654,000–1,989,000)
The Pan-American Health Organization (PAHO)	126,000 (101,000–158,000)	280,000 (228,000–347,000)	290,000 (241,000–357,000)	245,000 (201,000–315,000)	226,000 (200,000–297,000)	262,000 (223,000–319,000)	188,000 (158,000–229,000)	171,000 (142,000–208,000)	195,000 (164,000–235,000)	187,000 (159,000–230,000)	2,170,000 (1,820,000–2,692,000)
South East Asia regional office (SEARO)		84,100 (69,700–118,000)	220,000 (179,000–392,000)	197,000 (160,000–332,000)	192,000 (156,000–322,000)	99,500 (86,100–192,000)	65,600 (54,300–110,000)	68,200 (57,900–107,000)	80,400 (67,500–100,000)	89,600 (75,100–111,000)	1,096,000 (910,000–1,747,000)
Western Pacific regional office (WPRO)	910 (730–1,100)	173,000 (158,000–226,000)	135,000 (122,000–378,000)	105,000 (96,400–369,000)	108,000 (100,000–311,000)	81,400 (76,100–255,000)	116,000 (105,000–176,000)	134,000 (115,000–171,000)	124,000 (105,000–150,000)	124,000 (105,000–150,000)	1,101,000 (1,052,000–2,160,000)
**World Bank income group**	**2014**	**2015**	**2016**	**2017**	**2018**	**2019**	**2020**	**2021**	**2022**	**2023**	**2014-2023**
High income	130,000 (104,000–161,000)	510,000 (461,000–602,000)	512,000 (461,000–662,000)	489,000 (447,000–608,000)	475,000 (453,000–545,000)	470,000 (439,000–538,000)	336,000 (310,000–397,000)	289,000 (262,000–342,000)	291,000 (263,000–337,000)	301,000 (271,000–348,000)	3,803,000 (3,483,000–4,506,000)
Upper middle income		105,000 (90,600–138,000)	116,000 (107,000–278,000)	85,600 (82,300–306,000)	74,800 (72,200–333,000)	127,000 (120,000–323,000)	147,000 (139,000–205,000)	175,000 (156,000–210,000)	176,000 (158,000–209,000)	163,000 (147,000–199,000)	1,169,000 (1,144,000–2,156,000)
Lower middle income		1,145,000 (960,000–1,403,000)	944,000 (826,000–1,202,000)	1,058,000 (983,000–1,294,000)	819,000 (756,000–1,212,000)	2,071,000 (1,730,000–2,552,000)	281,000 (270,000–377,000)	238,000 (202,000–335,000)	279,000 (235,000–337,000)	328,000 (281,000–391,000)	7,163,000 (6,343,000–8,903,000)
Low income		9,200 (8,500–10,200)	7,500 (7,000–8,400)	11,400 (10,700–12,800)	12,700 (12,400–15,200)	4,300 (4,100–28,200)	32,300 (31,000–69,200)	27,400 (25,200–52,100)	32,800 (27,800–39,300)	11,000 (10,500–33,000)	149,000 (140,000–267,000)
**European Union**	**2014**	**2015**	**2016**	**2017**	**2018**	**2019**	**2020**	**2021**	**2022**	**2023**	**2014-2023**
European Union	1,300 (1,100–1,700)	127,000 (124,000–150,000)	128,000 (117,000–177,000)	149,000 (139,000–189,000)	149,000 (143,000–172,000)	126,000 (122,000–141,000)	70,100 (66,400–93,900)	58,800 (54,900–65,600)	58,900 (55,300–65,500)	62,800 (59,400–69,700)	930,000 (887,000–1,121,000)
**Continent**	**2014**	**2015**	**2016**	**2017**	**2018**	**2019**	**2020**	**2021**	**2022**	**2023**	**2014-2023**
Africa		839,000 (673,000–1,046,000)	356,000 (288,000–442,000)	499,000 (496,000–621,000)	252,000 (207,000–533,000)	1,608,000 (1,288,000–2,031,000)	56,300 (53,600–103,000)	34,300 (32,000–59,900)	42,700 (38,200–50,400)	21,000 (20,800–45,900)	3,708,000 (3,106,000–4,921,000)
Asia		504,000 (456,000–605,000)	744,000 (675,000–1,079,000)	694,000 (624,000–1,032,000)	702,000 (670,000–1,027,000)	611,000 (550,000–868,000)	426,000 (417,000–541,000)	403,000 (373,000–514,000)	427,000 (382,000–495,000)	480,000 (432,000–556,000)	4,991,000 (4,645,000–6,596,000)
Australia and New Zealand	910 (730–1,100)	4,000 (3,200–6,200)	34,600 (27,700–43,300)	22,500 (18,000–29,200)	16,000 (14,700–21,500)	14,800 (14,100–18,500)	9,200 (8,900–13,900)	7,400 (7,100–9,200)	5,900 (5,800–7,400)	6,200 (5,800–7,800)	121,000 (106,000–158,000)

(*continued on next page*)

Table 2. (*continued*)

WHO region	2014	2015	2016	2017	2018	2019	2020	2021	2022	2023	2014-2023
Europe	2,600 (2,200–3,100)	143,000 (140,000–167,000)	154,000 (143,000–205,000)	183,000 (175,000–227,000)	185,000 (181,000–213,000)	177,000 (173,000–221,000)	116,000 (112,000–159,000)	114,000 (107,000–126,000)	110,000 (106,000–122,000)	108,000 (101,000–121,000)	1,293,000 (1,254,000–1,530,000)
Latin America and the Caribbean		9,100 (7,600–11,100)	49,000 (40,500–60,500)	21,700 (18,600–43,300)	21,900 (19,400–49,300)	49,700 (41,900–62,400)	29,700 (25,400–35,600)	26,600 (22,900–33,100)	41,300 (35,700–48,900)	33,600 (30,400–44,600)	283,000 (246,000–386,000)
Northern America	126,000 (101,000–158,000)	271,000 (219,000–339,000)	242,000 (194,000–305,000)	223,000 (179,000–279,000)	204,000 (177,000–256,000)	212,000 (175,000–265,000)	158,000 (129,000–198,000)	144,000 (115,000–180,000)	153,000 (125,000–192,000)	153,000 (125,000–192,000)	1,888,000 (1,538,000–2,362,000)
**Global**	**2014**	**2015**	**2016**	**2017**	**2018**	**2019**	**2020**	**2021**	**2022**	**2023**	**2014-2023**
Global	130,000 (104,000–161,000)	1,770,000 (1,589,000–2,068,000)	1,748,000 (1,666,000–2,144,000)	1,777,000 (1,725,000–2,192,000)	1,484,000 (1,451,000–2,015,000)	2,731,000 (2,433,000–3,340,000)	796,000 (804,000–968,000)	729,000 (703,000–874,000)	780,000 (732,000–867,000)	802,000 (760,000–907,000)	12,748,000 (12,226,000–15,231,000)

AFRO, African region; DAAs, direct-acting antivirals; EMRO, Eastern Mediterranean region; EURO, European region; HCV, hepatitis C virus; PAHO, Pan American Health Organization; SEARO, Southeast Asian region; WPRO, Western Pacific region.

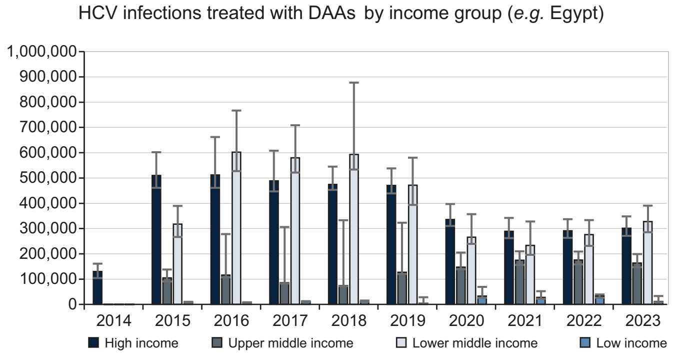

Fig. 2. Countries/regions accounting for 85% of all DAA treatments globally in 2014-2023. DAAs, direct-acting antivirals; EU, European Union.

Pakistan, the country with the second largest number of treated PLHCV after Egypt (Fig. 2), is a special case as more than 70% of all its HCV treatments were purchased in the private market. This model required the availability of treatments at an affordable price, demonstrating the importance of the private market to support the national elimination efforts. Pakistan has shown that at affordable prices, some portion of the population can afford to purchase the medicine on their own. Pakistan's treatment numbers did peak in 2018, but the Prime Minister's plan is underway to expand their national elimination program under the public health system.

The United States and the EU had the next largest number of DAA-treated infections (Fig. 2). In the United States, the number of treated cases peaked in 2015 shortly after their launch, while in the EU, the peak occurred in 2017 when nearly all member states removed treatment restrictions after price negotiation and reimbursement approval. The same pattern was observed in other HICs: a peak in treatment when DAAs were reimbursed, followed by a steady decline (Table 1).

The number of DAA-treated PLHCV in India (Fig. 2) was difficult to estimate due to exports of DAAs to other countries for sale in the private market by individuals. Initially, these reports were discounted, but a review of the national program and adjustments of sales in India's private market could only account for 90,000–194,000 treated PLHCV in years 2016-2019 (Table 1). The remaining DAAs, which could have treated an

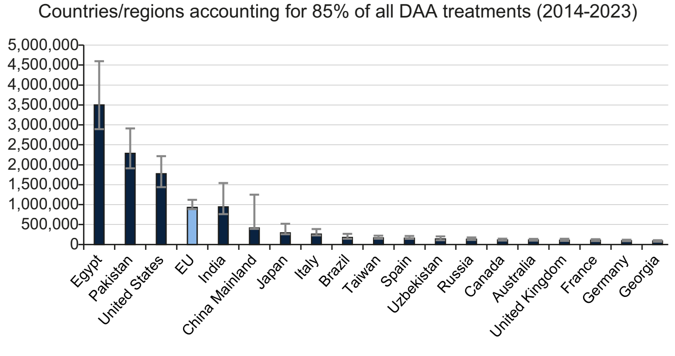

Fig. 3. Total PLHCV treated with DAAs by World Bank income group regions (excluding treatments in Egypt). DAAs, direct-acting antivirals; HCV, hepatitis C virus; PLHCV, people living with hepatitis C virus.

additional 463,000 PLHCV, were classified as "other" since their whereabouts could not be accounted for in India. DAA sales data for India was not available after 2019.

The number of DAA-treated PLHCV in China was also very difficult to estimate since locally manufactured treatment was being used for internal consumption. IQVIA DAA sales data were used to estimate the total number of treated PLHCV, but it led to a gross underestimation. We estimate that 93,000–95,000 PLHCV have been treated with DAAs annually since 2020. Other countries with a high number of treated PLHCV (Japan, Italy, Brazil, Spain, and Taiwan) all had funded national strategies in place, which explains their high numbers (Italy has the highest number of treated PLHCV in the EU) (Fig. 2).

Impact of price and reimbursement

The number of DAA-treated PLHCV in HICs peaked in 2016 (Fig. 3), when the prices of DAAs were near their peak, and the number of treated cases dropped by 47% while the prices of DAAs declined over the 2014-2023 period. In the EU countries, there was an immediate increase in the number of treated PLHCV as soon as DAA treatment for those with fibrosis scores of F0-F2 was reimbursed in 2017, with some countries (*e.g.*, the Netherlands) starting earlier. However, in HICs, there was no increase in DAA treatment as prices declined. These trends suggest that in HICs, price negotiations were important to reach the cost-effectiveness and reimbursement thresholds. Further price reductions were used to expand related activities, but the price reduction did not lead to an increase in the number of treated PLHCV. Thus, in HICs, a threshold price was needed for reimbursement, and treatment reimbursement itself was the key predictor of an increase in treatment.

On the other hand, the relationship between DAA prices was strongly correlated with the number of treatment initiations in low- and middle-income countries (LMICs). The number of treated PLHCV in upper middle-income countries increased as DAA prices dropped and countries like China, Russia, Malaysia, and Brazil continued to expand their treatment programs. The number of treated PLHCV also increased in low-income countries (Table 2) as the prices of DAAs dropped. In lower middle-income countries, a similar relationship was observed; however, this trend peaked in 2018 (Fig. 3) when the pool of diagnosed and motivated PLHCV who were able to pay for their treatment was depleted in Pakistan and India. These trends suggest that traditional price elasticity of demand applies in LMICs, where an increase in the number of treated PLHCV was observed with lowering prices until the initial warehoused pool of PLHCV was depleted.

Treatment in the absence of a screening program

The number of treated PLHCV stagnated/declined in every country/region irrespective of income level (Table 1). This trend was observed much faster in HICs, while in LMICs (*e.g.*, Pakistan and Indonesia), it took longer to manifest. This trend may be the result of the depletion of the pool of PLHCV waiting for treatment who can pay (in countries where PLHCV must pay out of pocket). The decline in the number of treatments has led to the conclusion, in some countries, that HCV prevalence must be lower than originally estimated. However, declining numbers of treated PLHCV may be more indicative of exhausting the efficacy of existing strategies rather than a lower HCV prevalence.

To achieve the WHO elimination targets, countries should consider expanding screening programs. This was best demonstrated in the Egyptian program, where adoption of a national screening campaign in 2019 resulted in a very large increase in treatment. In the United Kingdom, England initiated a wide-scale screening program to maintain their treatment numbers. Similarly, after Germany initiated a one-time screening of adults in 2022, an increase in the number of treated PLHCV was observed. The recommendations to screen adults for HCV in the United States also led to an increase in treatment initiations.

PLHCV segmentation

Countries are finding that treating 80% of all diagnosed PLHCV is difficult, as more recent studies suggest that one-third of diagnosed individuals are motivated to come in for a curative treatment within the first 1-3 years,[10,11] one-third can be connected to care via awareness and linkage to care programs,[12] and the last third will require additional programs to be linked to care. The low linkage to care in the general population may be explained by low awareness of HCV and its relation to liver cancer. A survey by the World Hepatitis Alliance found that only 42% of all respondents knew of a relationship between viral infection and liver cancer,[13] while a similar study in Brazil found that only 23% of the respondents attributed HCV infection to liver cancer.[14] Similarly, a study in Uzbekistan found that 42% of PLHCV lost to follow-up did not know their infection could lead to liver cancer.[15] Although screening for HCV is important to meet the WHO elimination targets, awareness programs will be needed to motivate PLHCV to seek treatment. In addition, linkage to care programs will be needed to bring in those who are less motivated to seek care. Studies have shown that it is possible to bring >30% of lost-to-follow-up PLHCV back into care through active linkage to care initiatives.[12,16,17]

Relevant lessons for other liver diseases

An analysis of HCV treatment is timely as several products are in development for the functional cure of HBV. In addition, there is already a product on the market for the treatment of HDV, while several pipeline products are targeting HDV treatment. The same concerns that were present before the launch of DAAs are being voiced again. Will the new treatments put an undue burden on national healthcare budgets? Are there

enough specialists to treat the new wave of patients coming in for treatment as these products launch? And how will we deal with the large burden of these diseases in LMICs? The historical trends in HCV treatment can provide lessons that could be applicable to the pipeline therapies.

When DAAs launched, there was much concern about health systems being severely economically challenged due to their high prices and the instant inflow of PLHCV to be treated. The history of DAA treatment seems to suggest that in HICs, the national reimbursement agencies are competent and only approve treatments once they are cost-effective. In LMICs, price does play an important role, and treatments will remain limited until prices drop sufficiently.

Another concern raised before the launch of DAAs was that countries may not have enough specialists to treat the large inflow of new patients as new therapies are launched. This can apply to HBV as well. The HCV experience has taught us that only a fraction of all diagnosed patients will come in for treatment, and in fact, much effort (screening, awareness, and linkage to care) is needed to bring in patients for treatment. Treatment simplification can also help engage other healthcare professionals (general practitioners and nurses) to treat a portion of the population.

There is a key distinction between HCV and HBV (and HDV) that is worth noting. The HCV diagnosis rate was already much higher at the time when DAAs were launched. In comparison, HBV diagnosis rates are close to half those of HCV, and the HDV diagnosis rate is low.[4,18] The launch of new therapies for HBV and HDV will require collaborations between hospitals, major clinics, universities, and national governments to screen for these diseases, but more importantly, countries need national registries to keep track of the positive and negative test results for linkage to care and retention in care programs once new treatments are available.

Study limitations

There were several challenges with our study. Our methodology was highly dependent on national reporting. Biases in these estimates would carry over to our analysis as well. Unit sales data from IQVIA were only reliable in HICs. In LMICs, this data captured only specific channels (*e.g.* hospital sales) and had to be adjusted for the portion of total sales represented by those channels to generate more accurate treatment estimates.

The review of companies' presentations to investors was helpful in the years 2014–2019 when companies reported the number of treated PLHCV for their products and the entire market in the United States and the EU. For our study, we did not purchase and analyze financial analysts' reports.

In LMICs, the majority of HCV treatment occurred at a handful of tertiary centers. Physician interviews and a review of hospital records provided a reasonable estimate of the total number of treated PLHCV in the country when compared to drug sales data. This approach, however, was less successful in HICs where PLHCV had access through multiple channels (tertiary hospitals, clinics, prisons, harm reduction centers, and pharmacies).

Export/import data were analyzed by country, but they significantly underestimated the number of bottles shipped to each country when compared to the data reported by generic manufacturers to patent holders. There were also large discrepancies between the treated PLHCV estimated via sales figures, the national programs, and those provided by national experts in LMICs. A significant amount of DAAs purchased (est. more than 15%) in LMICs appear to be expiring, presumably due to the products not being distributed to hospitals, complex treatment algorithms, a shortage of related supplies (*e.g.*, PCR reagents, antibody tests, etc.), and an insufficient number of diagnosed PLHCV coming in for treatment. The upper bound of our UIs did include purchases that may have expired unless the country specifically notified us of the volume of products that had expired. In that case, the expired products were subtracted from our estimates.

The "other" group captured treated PLHCV who could not be allocated to a specific country. The example of India was discussed above, but there were also shipments of generic DAAs to European and Middle Eastern countries that were clearly used in programs run by international agencies like Médecins Sans Frontières. Those shipments were all captured under "other". Thus, there could be some double counting of treated PLHCV when the countries being serviced by these organizations reported their estimates separately.

Another limitation was not taking into consideration a longer duration of therapy (with or without ribavirin) for treatment of patients with cirrhosis or retreatment of DAA treatment failures. Doing so would result in a lower number of calculated treated PLHCV, as they would require an additional number of bottles to treat the same individual. However, only 19% of the total estimates came from analysis of DAA sales data, and if 25% of all DAAs sold in these countries used double the duration of treatment for the above populations, our overall estimates would be 2.5% lower. This is well within our confidence UIs.

Our analysis also did not take into consideration retreatment of reinfections. Doing so would result in a lower number of calculated treated PLHCV, as they would be counted multiple times if the national registry is only reporting treatment initiation. This is important in countries where the epidemic is predominantly driven by injecting drug use and access to needle and syringe programs, or opioid substitution therapy is not available or is limited. Although reinfection rates are relatively high (6.6/100 person-years; 95% CI 3.4-12.7) in these settings,[19] the percentage of total PLHCV who are actively injecting, are reinfected, and are subsequently treated will be small and well within our confidence UIs.

Finally, the adjustment of bottles sold to treat PLHCV assumed 8-12 weeks of treatment, depending on the regimen. However, PLHCV may not be 100% treatment compliant. This would result in a higher number of HCV treatments than shown here.

Conclusions

In the last 10 years, DAAs have cured 21% of the infected population worldwide and 30% of the target population (taking

into consideration 90% diagnosis and 80% treatment targets). However, 7 years remain to treat the remaining 70% of the target population. To accomplish this, expanded access, active HCV screening, and linkage to care may be required.

Abbreviations

DAAs, direct-acting antivirals; EU, European Union; HBV, hepatitis B virus; HCV, hepatitis C virus; HDV, hepatitis delta virus; HICs, high-income countries; LMICs, low- and middle-income countries; PLHCV, people living with hepatitis C virus; WHO, World Health Organization; UI, uncertainty interval.

Financial support

This project was made possible by the Polaris Observatory, which was fully funded by the John C. Martin Foundation (G62), a privately funded foundation, in 2024. Since inception, Polaris has received 79% of its funding from the John C. Martin Foundation (G01, G02, G11, G24, G39, & G62), 8% from private donors, 4% from Gilead Sciences (investigator-sponsored research grant IN-US-987-5808), 3% from the Zeshan Foundation (2021-0101-1-CDA-HEP-10), 1.5% from AbbVie, 1.5% from the Hepatitis Fund, 1.3% from the World Health Organization, and the remaining from other donors. The funders had no role in the study design, data collection, data analysis, data interpretation, or preparation of the manuscript.

Conflict of interest

Please refer to the accompanying ICMJE disclosure forms for further details.

Authors' contributions

HR conceived the study, designed the methodology, and was responsible for the project administration. HR, DMR-S, and IG conducted the formal analysis. HR wrote the original draft and updated it after feedback from all first authors (IW, HQ, LAK, ASD, SA, JT, JVL, DLB). HR, IG, DMR-R, ASV, SH, and KR-S had access to the underlying data and verified the data. All authors curated and validated the data, as well as reviewed and edited the manuscript. All authors had full access to the data for their country and accepted the responsibility to submit their data for publication.

Data availability statement

All data presented in this article is accessible through the Polaris Observatory website (https://cdafound.org/polaris/database-query/).

Acknowledgements

We want to thank Sarah Blach, who led the HCV efforts at the Polaris Observatory until 2022. We also want to acknowledge all Polaris Observatory collaborators, who over the last 10 years have contributed to our data collection, analysis, and modeling. This would not have been possible without their support. We are also grateful to Gilead Sciences, AbbVie, Ferozsons Laboratories, and their national affiliates for sharing their estimates of the number of treated PLHCV. Finally, we are grateful to Ken Pasini and Randy Wilp for teaching us how to convert drug sales data to treated PLHCV, which is more of an art than a science.

Supplementary data

Supplementary data to this article can be found online at https://doi.org/10.1016/j.jhep.2025.01.013.

References

[1] World Health Organization. Global hepatitis report 2024: action for access in low- and middle-income countries. Geneva: World Health Organization; 2024.
[2] Assembly WHOS-NWH. Draft global health sector strategies viral hepatitis 2016-2021. 2016.
[3] WHO. Combating hepatitis B and C to reach elimination by 2030. Geneva, Switzerland: WHO; May 2016 2016.
[4] Blach S, Terrault NA, Tacke F, et al. Global change in hepatitis C virus prevalence and cascade of care between 2015 and 2020: a modelling study. Lancet Gastroenterol Hepatol 2022;7(5):396–415.
[5] Cui F, Blach S, Manzengo Mingiedi C, et al. Global reporting of progress towards elimination of hepatitis B and hepatitis C. Lancet Gastroenterol Hepatol 2023;8(4):332–342.
[6] Center for Disease Analysis Foundation. List of countries which have access to generic direct-acting antivirals for hepatitis C, 2024. https://cdafound.org/gpro/list-of-hcv-access-countries/. [Accessed 13 March 2024]. 2024.
[7] Wang H, Dwyer-Lindgren L, Lofgren KT, et al. Age-specific and sex-specific mortality in 187 countries, 1970-2010: a systematic analysis for the Global Burden of Disease Study 2010. Lancet. 2012; 380(9859):2071–2094.
[8] Gountas I, Sypsa V, Anagnostou O, et al. Treatment and primary prevention in people who inject drugs for chronic hepatitis C infection: is elimination possible in a high-prevalence setting? Addiction 2017; 112(7):1290–1299.
[9] Waked I, Esmat G, Elsharkawy A, et al. Screening and treatment program to eliminate hepatitis C in Egypt. New Engl J Med 2020;382(12): 1166–1174.
[10] Aleman S, Soderholm J, Busch K, et al. Frequent loss to follow-up after diagnosis of hepatitis C virus infection: a barrier towards the elimination of hepatitis C virus. Liver Int 2020;40(8):1832–1840.
[11] Thompson WW, Symum H, Sandul A, et al. Vital signs: hepatitis C treatment among insured adults - United States, 2019-2020. MMWR Morb Mortal Wkly Rep 2022;71(32):1011–1017.
[12] Wyatt B, Perumalswami PV, Mageras A, et al. A digital case-finding algorithm for diagnosed but untreated hepatitis C: a tool for increasing linkage to treatment and cure. Hepatology 2021;74(6):2974–2987.
[13] James C, Hicks J, Smith A. The awareness of the link between liver cancer and hepatitis as a motivation for action – results of a globally representative survey. EASL Liver Cancer Summit 2024 2024. Rotterdam, Netherlands.
[14] Bittencourt PL, Codes L, Cesar HF, et al. Public knowledge and attitudes toward liver diseases and liver cancer in the Brazilian population: a cross sectional study. Lancet Reg Health Am 2023;23:100531.
[15] Musabaev E, Estes C, Sadirova S, et al. Viral hepatitis elimination challenges in low- and middle-income countries-Uzbekistan Hepatitis Elimination Program (UHEP). Liver Int 2023;43(4):773–784.
[16] Chen CJ, Huang YH, Hsu CW, et al. Hepatitis C micro-elimination through the retrieval strategy of patients lost to follow-up. BMC Gastroenterol 2023;23(1):40.
[17] Drose S, Hansen JF, Roge BT, et al. Retrieval of patients with hepatitis C who were lost to follow-up in Southern Denmark. Infect Dis (Lond) 2023;55(5):361–369.
[18] Razavi-Shearer D, Razavi H. Global prevalence of hepatitis B virus infection and prevention of mother-to-child transmission - authors' reply. Lancet Gastroenterol Hepatol 2018;3(9):599.
[19] Hajarizadeh B, Cunningham EB, Reid H, et al. Direct-acting antiviral treatment for hepatitis C among people who use or inject drugs: a systematic review and meta-analysis. Lancet Gastroenterol Hepatol 2018;3(11): 754–767.
[20] Ministry of Health Argentina. Cumulative number of patients treated. In. Buenos Aires, Argentina2020.
[21] The Kirby Institute. Monitoring hepatitis C treatment uptake in Australia. Sydney, NSW, Australia: The Kirby Institute; 2021.
[22] Burnet Institute and Kirby Institute. Australia's progress towards hepatitis C elimination: annual report 2023. 2023.
[23] Hajarizadeh B, Grebely J, Matthews GV, et al. Uptake of direct-acting antiviral treatment for chronic hepatitis C in Australia. J Viral Hepat 2018;25(6):640–648.

[24] Busschots D, Ho E, Blach S, et al. Ten years countdown to hepatitis C elimination in Belgium: an extensive mathematical modelling approach. 2021. *Ahead of print*.
[25] Brasil Ministerio da Saude. Monitoramento. 2021.
[26] National Health Insurance Fund (NHIF). Annual number of HCV patients treated. 2021.
[27] Flisiak R, Zarebska-Michaluk D, Ciupkeviciene E, et al. HCV elimination in central Europe with particular emphasis on microelimination in prisons. Viruses 2022;14(3).
[28] Médecins Sans Frontières (MSF). Cambodia hep C: seology, PCR and treatment data, 2016-2020. In. MSF, trans. Phnom-Penh2021.
[29] Nim N. Adding HCV diagnosis and treatment data from NCHADS and SHCH. In: Dugan E, ed. Phnom Penh, Cambodia2021.
[30] Popovic N, Williams A, Perinet S, et al. National Hepatitis C estimates: incidence, prevalence, undiagnosed proportion and treatment, Canada, 2019. Can Commun Dis Rep = Releve des maladies transmissibles au Can 2022;48(11–12):540–549.
[31] Fondo Colombiano de Enfermedades de Alto Costo CdACC. Situación de la hepatitis C crónica en los regímenes subsidiado y contributivo de Colombia 2020. 2021. Bogota, D.C.
[32] Statistics on discounted medicines. Estonian health insurance Fund, 2021. https://www.haigekassa.ee/haigekassa/finantsnaitajad/soodusravimite-statistika. [Accessed 2 June 2021].
[33] Hungarian Society of Gastroenterology, Section H. Hepatitis registry. https://hepreg.hu/pages/login.php.
[34] Ministry of Health Republic of Indonesia. Hepatitis C information system, 2023. https://sihepi.kemkes.go.id/hepc/dashboard/dashboard_main.php; 2023. 2/14/2024, 2024.
[35] Agenzia Italiana del Farmaco. Registri AIFA per il monitoraggio dei farmaci anti-HCV, 2023. https://www.aifa.gov.it/en/aggiornamento-epatite-c; 2023.
[36] Centre for Disease Prevention and Control of Latvia. [Infectious diseases statistics 2002-2019], 2020. http://www.spkc.gov.lv/infekcijas-slimibu-statistika/; 2020. 1/5/2021, 2021.
[37] Lithuania National Health Insurance Fund. Number of patients treated for HCV annually through 2020. 2021. Data provided by Dr. Valentina Liakina.
[38] Yuswan F. Hepatitis C surveillance Malaysia. Pharmaceutical Service Program Ministry of Health Malaysia; 2021.
[39] Netherlands HIot. GIPdatabank 2024. https://www.gipdatabank.nl/databank?infotype=g&label=00-totaal&tabel=B_01-basis&geg=gebr&item=J05AP. 2/14/2024, 2024.
[40] Antivirals for treatment of HCV infections, number of users, 2004 - 2020. Norwegian Prescription Database, The Norwegian Institute of Public Health (NIPH); 2021. http://www.norpd.no/Prevalens.aspx. [Accessed 27 February 2024].
[41] Ministry of National Health Services. Data from four provincial hepatitis control programs 2015-2019. 2019. Islamabad, Pakistan.
[42] Flisiak R, Zarebska-Michaluk D, Janczewska E, et al. Changes in characteristics of HCV infected patients treated in Poland between 2015 and 2017 EpiTer 2 study. J Viral Hepat 2018;25:170.
[43] Debu M. Data from the national HCV screening program in Romania. In: Center for disease analysis foundation; 2020. Bucharest, Romania: Association of Patients with Liver Diseases in Romania (APAH-RO).
[44] Ministry of Health Republic of Rwanda. Rwanda national HIV and viral hepatitis annual report 2017-2018. Kigali, Rwanda2019.
[45] National health information center (NCZI), 2024. https://www.nczisk.sk/en/Pages/default.aspx; 2024. 2024, 3/1/2024.
[46] Spain Ministry of Health. Gobierno de España. Ministerio de Sanidad. Secrtaría de Estado de Sanidad [Number of patients who start treatment for chronic hepatitis C with direct-acting antivirals], 2023. https://www.sanidad.gob.es/areas/farmacia/publicaciones/planOptimizacion/docs/hepatitisC/Pacientes_Tratados_Hasta_30_09_2023.pdf. [Accessed 10 March 2024].
[47] Sweden Statistics. Statistics database for medicines, 2024. https://sdb.socialstyrelsen.se/if_lak/val.aspx; 2024. 2/14/2024, 2024.
[48] InfCare hepatitis. InfCareHepatit, 2024. https://www.infcarehepatit.se/about-infcarehepatit; 2024. 1/1/2024, 2024.
[49] UK Health Security Agency. Research and analysis hepatitis C in England 2023, 2024. https://www.gov.uk/government/publications/hepatitis-c-in-the-uk/hepatitis-c-in-england-2023; 2024.
[50] UK Health Security Agency. Hepatitis C in the UK 2023 - working to eliminate hepatitis C as a public health threat, 2023. https://assets.publishing.service.gov.uk/government/uploads/system/uploads/attachment_data/file/1133731/hepatitis-c-in-the-UK-2023.pdf; 2023.
[51] Public Health Scotland. Surveillance of hepatitis C in Scotland, 2023, update: progress on elimination of hepatitis C as a major public health concern, 2023. https://publichealthscotland.scot/media/24025/hcvsurveillancereport2023_final.pdf. [Accessed 2 July 2024].
[52] NHS Wales. Prevention, diagnosis and treatment of blood borne viruses in Wales: hepatitis B, hepatitis C and HIV, Annual report 2023, 2023. https://phw.nhs.wales/publications/publications1/prevention-diagnosis-and-treatment-of-blood-borne-viruses-in-wales-hepatitis-b-hepatitis-c-and-hiv/; 2023.

Keywords: Polaris Observatory; Regions; Global; WHO regions; World Bank regions.
Received 3 July 2024; received in revised form 9 January 2025; accepted 10 January 2025; available online 4 February 2025

Appendix.

The following authors in bold share first authorship: **Homie A Razavi, Imam Waked, Huma Qureshi, Loreta A Kondili, Ann-Sofi Duberg, Soo Aleman, Junko Tanaka, Jeffrey V Lazarus, and Daniel Low-Beer**.

Authors:

Homie A Razavi[1], Imam Waked[2], Huma Qureshi[3], Loreta A Kondili[4,5], Ann-Sofi Duberg[6], Soo Aleman[7], Junko Tanaka[8], Jeffrey V Lazarus[9,10], Daniel Low-Beer[11], Zaigham Abbas[12], Antoine Abou Rached[13], Alessio Aghemo[14,15], Inka Aho[16], Ulus S Akarca[17], Said A Al-Busafi[18], Waleed K Al-Hamoudi[19], Khalid Al-Naamani[20], Ahmed Sabry Alaama[21], Manahil M Aldar[22], Mohammed Alghamdi[23], Monica Alonso Gonzalez[24], Haleema Alserehi[25], Anil C Anand[26], Tarik Asselah[27], Abdullah M Assiri[28], Kostas Athanasakis[29], Rita Atugonza[30], Ziv Ben-Ari[31,32], Thomas Berg[33], Carlos E Brandão-Mello[34,35], Ashley S M Brown[36], Kimberly A Brown[37], Robert S Brown, Jr[38], Philip Bruggmann[39], Maurizia R Brunetto[40,41], Maria Buti[42], Hugo Cheinquer[43], Peer Brehm Christensen[44,45], Vladimir Chulanov[46,47], Laura E Cisneros Garza[48], Carla S Coffin[49], Nicola Coppola[50], Antonio Craxi[51], Javier Crespo[52,53], Fuqiang Cui[54], Olav Dalgard[55], Alethse De La Torre[56], Victor De Ledinghen[57], Douglas Dieterich[58], Sylvia Drazilova[59], Jean-François Dufour[60], Mohamed El-Kassas[61], Mohammed Elbadri[62], Gamal Esmat[63], Rafael Esteban Mur[64], Brandon Eurich[1], Diana Faini[65], Paulo R A Ferreira[66], Robert Flisiak[67], Sona Frankova[68], Giovanni B Gaeta[69], Ivane Gamkrelidze[1], Edward J Gane[70], Virginia Garcia[71], Javier García-Samaniego[72], Manik Gemilyan[73], Magnus Gottfredsson[74,75], Michael Gschwantler[76,77], Ana P M Gurski[78], Behzad Hajarizadeh[79], Saeed S Hamid[80], Angelos Hatzakis[81], Julian Hercun[82], Ivana Hockicková[83], Jee-Fu Huang[84], Bela Hunyady[85], Sharon J Hutchinson[86,87], Naoko Ishikawa[11], Kiyohiko Izumi[88], Antonio Izzi[89], Martin Janicko[59], Peter Jarcuska[59], Agita Jeruma[90,91], Asgeir Johannessen[92], Kulpash S Kaliaskarova[93], Jia-Horng Kao[94], Knut B Kielland[95], Nicolas Kodjoh[96], Shyamasundaran Kottilil[97], Pavol Kristian[83], Paul Y Kwo[98], Martin Lagging[99,100], Hilton Lam[101], Pablo Lázaro[102], Mei-Hsuan Lee[103], Sabela Lens[104], Valentina Liakina[105,106], Young-Suk Lim[107], Michael Makara[108], Michael Manns[109], Casimir Mingiedi Manzengo[110], Sadik Memon[111], Maria Cássia Mendes-Correa[112], Vincenzo Messina[113], Håvard Midgard[114], Niamh Murphy[115], Erkin Musabaev[116], Marcelo C M Naveira[117], Helen Nde[1], Francesco Negro[118], Nirada Nim[119], Ponsiano Ocama[120], Sigurdur Olafsson[121], Casimir E Omuemu[122], Javier Pamplona[123], Calvin Q Pan[124], George V Papatheodoridis[125], Nikolay Pimenov[46,126], Hossein Poustchi[127], Maria Giovanna Quaranta[128], Alnoor Ramji[129], Henna Rautiainen[130], Devin M Razavi-Shearer[1], Kathryn Razavi-Shearer[1], Ezequiel Ridruejo[131], Cielo Y Ríos-Hincapié[132], Shakhlo Sadirova[133], Faisal M Sanai[134], Christoph Sarrazin[135,136], Gulya Sarybayeva[137], Ivan Schréter[138], Carole Seguin-Devaux[139], Leandro S Sereno[24], Gamal Shiha[140,141], Josie Smith[142], Riham Soliman[143], Mark W Sonderup[144], C Wendy Spearman[144], Rudolf E Stauber[145], Catherine A M Stedman[146,147], Vana Sypsa[81], Frank Tacke[148], Norah A Terrault[149], Ieva Tolmane[150,151], Berend Van Welzen[152], Alexis S Voeller[1], Yasir Waheed[153,154], Carolyn Wallace[1], Robert N Whittaker[155], Vincent W-S Wong[156,157], Magdalena Ydreborg[158], Kakharman Yesmembetov[159,160], Ming-Lung Yu[161,162,163], Stefan Zeuzem[164], Eli Zuckerman[165].

[1]Center for Disease Analysis Foundation, Lafayette, United States; [2]Hepatology, National Liver Institute, Shebeen El Kom, Egypt; [3]Gastroenterologist, Focal Point Hepatitis, Ministry of National Health Services, Regulations and Coordination, Islamabad, Pakistan; [4]National Center for Global Health, Istituto Superiore di Sanita, Rome, Italy; [5]UniCamillus-Saint Camillus International University of Health Sciences, Rome, Italy; [6]Department of Infectious Diseases, Faculty of Medicine and Health, Örebro University, Örebro, Sweden; [7]Department of Infectious Diseases, Karolinska University Hospital, Stockholm, Sweden; [8]Epidemiology, Infectious Disease Control and Prevention, Hiroshima University, Hiroshima, Japan; [9]CUNY Graduate School of Public Health and Health Policy, New York City, United States; [10]Barcelona Institute for Global Health (ISGlobal), Hospital Clínic, University of Barcelona, Barcelona, Spain; [11]Global Hepatitis Programme, World Health Organization, Geneva, Switzerland; [12]Hepatogastroenterology, Dr. Ziauddin University Hospital, Karachi, Pakistan; [13]Internal Medicine - Gastroenterology, Faculty of Medical Sciences, Lebanese University, Beirut, Lebanon; [14]Department of Biomedical Sciences, Humanitas University, Pieve Emanuele, Italy; [15]Department of Gastroenterology, Humanitas Research Hospital IRCCS, Rozzano, Italy; [16]Infectious Diseases, Helsinki University Hospital, Helsinki, Finland; [17]Department of Gastroenterology, Ege University, Medical School, Izmir, Turkey; [18]Medicine, College of Medicine and Health Sciences, Sultan Qaboos University, AlKhoudh, Oman; [19]Department of Medicine, King Saud University, Riyadh, Saudi Arabia; [20]Internal Medicine, Division of Gastroenterology and Hepatology, Armed Forces Hospital, Muscat, Oman; [21]World Health Organization Regional Office for the Eastern Mediterranean, Cairo, Egypt; [22]Viral Hepatitis Control Program, Ministry of Health, Ryiadh, Saudi Arabia; [23]Gastroenterology Unit, Department of Medicine, King Fahad Military Medical Complex, Dhahran, Saudi Arabia; [24]PAHO/WHO, Washington DC, United States; [25]Public Health, MOH, Riyadh, Saudi Arabia; [26]Gastroenterology & Hepatology, Kalinga Institute of Medical Sciences, Bhubaneswar, India; [27]Hepatology Department, Université de Paris-Cité, Hôpital Beaujon, AP-HP, INSERM UMR, Paris, France; [28]Public Health Department, Ministry of Health, Riyadh, Saudi Arabia; [29]Department of Public Health Policy, University of West Attica, Athens, Greece; [30]Ministry of Health, Kampala, Uganda; [31]Liver Medicine Center of Excellence, Assuta medical Centers, Tel Aviv, Israel; [32]Sackler School of Medicine, Tel-Aviv University, Tel-Aviv, Israel; [33]Division of Hepatology, Department of Medicine II, Leipzig University Medical Center, Leipzig, Germany; [34]Internal Medicine & Gastroenterology, University of Rio de Janeiro, Rio de Janeiro, Brazil; [35]Clinica de Doenças do Fígado, Rio de Janeiro, Brazil; [36]Department of Hepatology, Imperial College Healthcare NHS Trust, London, United Kingdom; [37]Department of Medicine, Henry Ford Hospital, Detroit, United States; [38]Medicine, Weill Cornell Medicine, New York, United States; [39]Arud Centre for Addiction Medicine, Zürich, Switzerland; [40]Clinical and Experimental Medicine, University of Pisa, Pisa, Italy; [41]Hepatology Unit, University Hospital of Pisa, Pisa, Italy; [42]Liver Unit, Hospital Universitari Vall d Hebron and CIBEREHD del Insituto Carlos III. Universidad Autonoma de Barcelona, Barcelona, Spain; [43]Department of Gastroenterology and Hepatology, Hospital de Clinicas de Porto Alegre (HCPA), Porto Alegre, Brazil; [44]Department of Infectious Diseases, Odense University

Hospital, Odense, Denmark; [45]Department of Clinical Research, University of Southern Denmark, Odense, Denmark; [46]National Medical Research Center of Phthisiopulmonology & Infectious Diseases, Moscow, Russia; [47]Department of Infectious Diseases, I.M. Sechenov First Moscow State Medical University, Sechenov University, Moscow, Russia; [48]Department of Hepatology, Hospital Christus Muguerza Alta Especialidad, Monterrey, Mexico; [49]Medicine/Microbiology and Infectious Diseases Department, Cumming School of Medicine, University of Calgary, Calgary, Canada; [50]Mental health and Public medicine, University of Campania, Naples, Italy; [51]PROMISE, School of Medicine, University of Palermo, Palermo, Italy; [52]Gastroenterology and Hepatology Department, Clinical and Translational Research in Digestive Diseases, Valdecilla Research Institute (IDIVAL), Marqués de Valdecilla University Hospital, Santander, Spain; [53]School of Medicine, University of Cantabria, Santander, Spain; [54]School of Public Health, Peking University, Beijing, China's Mainland; [55]Department of Infectious Diseases, Akershus University Hospital, Lørenskog, Norway; [56]Please Enter, Please Enter, Please Enter, Mexico; [57]Service d'hepatologie et de transplantation hepatique, CHU, Bordeaux, France; [58]Institute for Liver Medicine, Icahn School of Medicine at Mount Sinai, New York, United States; [59]2nd Dept of Internal Medicine, PJ Safarik University, Faculty of Medicine and L Pasteur University Hospital, Kosice, Slovakia; [60]Centre des Maladies Digestives, Lausanne, Switzerland; [61]Endemic Medicine Department, Helwan University, Cairo, Egypt; [62]Gastroenterology Department, Hamad Medical Corporation, Doha, Qatar; [63]Endemic medicine department, Cairo University Hospitals, Cairo, Egypt; [64]Department of Hepatology, Hospital Universitario Vall d'Hebron, Barcelona, Spain; [65]Global Hepatitis Programme, WHO, Geneva, Switzerland; [66]Division of Infectious Disease, Federal University of São Paulo, São Paulo, Brazil; [67]Department of Infectious Diseases and Hepatology, Medical University of Bialystok, Bialystok, Poland; [68]Department of Hepatogastroenterology, Institute for Clinical and Experimental Medicine, Prague, Czechia; [69]Infectious Diseases, University Vanvitelli, Napoli, Italy; [70]New Zealand Liver Transplant Unit, Auckland City Hospital, Auckland, New Zealand; [71]Gastroenterology Professor at Universidad Autonoma de Santo Domingo/Doctor at Centro Endoscopico Digestivo Integral/Retire doctor from the Health Ministry in the Dominican Republic, Santo Domingo, Dominican Republic; [72]Liver Unit, Hospital Universitario La Paz, CIBERehd/IdiPAZ. Universidad Autónoma de Madrid, Madrid, Spain; [73]Department of Gastroenterology and Hepatology, Yerevan State Medical University, Yerevan, Armenia; [74]Departments of science and infectious diseases, Landspitali University Hospital, Reykjavík, Iceland; [75]Faculty of Medicine, University of Iceland, Reykjavík, Iceland; [76]Department of Internal Medicine IV, Klinik Ottakring, Vienna, Austria; [77]Sigmund Freud University, Vienna, Austria; [78]Department of HIV/Aids, Tuberculosis, Viral Hepatitis and Sexually Transmitted Infections, Ministry of Health, Brasilia, Brazil; [79]The Kirby Institute, UNSW, Sydney, Australia; [80]Medicine, Aga Khan University, Karachi, Pakistan; [81]Department of Hygiene, Epidemiology and Medical Statistics, Medical School, National and Kapodistrian University of Athens, Athens, Greece; [82]Liver Unit, Centre hospitalier de l'Universite de Montreal, Montreal, Canada; [83]Department of Infectology and Travel Medicine, P.J. Safarik University, Faculty of Medicine and L. Pasteur University Hospital, Kosice, Slovakia; [84]Hepatobiliary Division, Department of Internal Medicine and Hepatitis Center, Kaohsiung Medical University Hospital, Kaohsiung Medical University, Kaohsiung, Taiwan; [85]Division of Gastroenterology, First Department of Medicine, Clinical Centre, University of Pecs, Pécs, Hungary; [86]School of Health and Life Sciences, Glasgow Caledonian University, Glasgow, United Kingdom; [87]Clinical and Protecting Health Directorate, Public Health Scotland, Glasgow, United Kingdom; [88]Division of Programmes for Disease Control, World Health Organization Regional Office for the Western Pacific, Manila, Philippines; [89]Department of Infectious Diseases and Emergency Infectious Disease, Division of Highly Contagious Infections Diseases, "D. Cotugno" hospital, Naples, Italy; [90]Riga East University hospital, Riga, Latvia; [91]Faculty of Medicine, Riga Stradins University, Riga, Latvia; [92]Vestfold Hospital Trust, Tonsberg, Norway; [93]National Research Oncology Center, Astana, Kazakhstan; [94]Hepatitis Research Center, National Taiwan University Hospital, Taipei, Taiwan; [95]Research Center for Substance Use Disorders and Mental Illness, Innlandet Hospital Trust, Brumunddal, Norway; [96]Faculté des Sciences de la Santé, Université d'Abomey Calavi, Cotonou, Benin; [97]Institute of Human Virology, University of Maryland School of Medicine, Baltimore, United States; [98]Medicine, Stanford University School of Medicine, Redwood City, United States; [99]Department of Infectious Diseases/Virology, Institute of Biomedicine, Sahlgrenska Academy, University of Gothenburg, Gothenburg, Sweden; [100]Department of Clinical Microbiology, Sahlgrenska University Hospital, Region Västra Götaland, Gothenburg, Sweden; [101]Institute of Health Policy and Development Studies, University of the Philippines, Manila, Philippines; [102]Independent Health Services Researcher, Madrid, Spain; [103]Institute of Clinical Medicine, National Yang Ming Chiao Tung University, Taipei, Taiwan; [104]Liver Unit, Hospital Clinic, IDIBAPS, University of Barcelona, CIBERehd, Barcelona, Spain; [105]Clinic of Gastroenterology, Nephrourology and Surgery, Institute of Clinical Medicine, Vilnius University, Faculty of Medicine, Vilnius, Lithuania; [106]Department of Chemistry and Bioengineering, VILNIUS TECH, Faculty of Fundamental Sciences, Vilnius, Lithuania; [107]Gastroenterology, Asan Medical Center, University of Ulsan College of Medicine, Seoul, Korea, Republic of; [108]Central Hospital of Southern Pest, National Institute of Hematology and Infectious Diseases, Budapest, Hungary; [109]Dept. of Gastroenterology, Hepatology and Endocrinology, Hannover Medical School, Hannover, Germany; [110]HIV/Aids, Tuberculosis and Hepatitis, WHO, Ouagadougou, Burkina Faso; [111]Gastroenterology, Asian Institute of Medical Sciences (AIMS), Hyderabad, Pakistan; [112]Department of Infectious Diseases, School of Medicine, University of São Paulo, São Paulo, SP, Br, Sao Paulo, Brazil; [113]Department of Infectious Diseases and Viral Hepatitis, Azienda Ospedaliera Sant'Anna e San Sebastiano di Caserta, Caserta, Italy; [114]Department of Gastroenterology, Oslo University Hospital, Oslo, Norway; [115]HSE, Health Protection Surveillance Centre, Dublin, Ireland; [116]Virology, Research Institute of Virology, Tashkent, Uzbekistan; [117]Communicable Diseases, Environment and Health, World Health Organization Regional Office for Europe, Copenhagen, Denmark; [118]Medicine, University of Geneva, Geneva, Switzerland; [119]CD team, WHO, Phnom Penh, Cambodia; [120]Internal Medicine, Makerere University, Kampala, Uganda; [121]Department of Gastroenterology and Hepatology,

Landspitali - National University Hospital of Iceland, Reykjavik, Iceland; [122]Department of Medicine, University of Benin, Benin City, Nigeria; [123]Hospital Santa Caterina, Salt, Girona, Spain; [124]Division of Gastroenterology and Hepatology, Department of Medicine, NYU Langone Health, NYU Grossman School of Medicine, New York, United States; [125]First Department of Gastroenterology, Medical School, National and Kapodistrian University of Athens, Athens, Greece; [126]Martsinovsky Institute of Medical Parasitology, Tropical and Vector-Borne Diseases, I.M. Sechenov First Moscow State Medical University (Sechenov University), Moscow, Russia; [127]Digestive Diseases Research Institute, Tehran University of Medical Sciences, Tehran, Iran; [128]Center for Global Health, Istituto Superiore di Sanità, Rome, Italy; [129]Department of Medicine, University of British Columbia, Vancouver, Canada; [130]Specialist in Gastroenterology and Internal Medicine, Helsinki University Hospital Abdominal Center, Helsinki, Finland; [131]Hepatology Section, Department of Medicine., Centro de Educación Médica e Investigaciones Clínicas Norberto Quirno “CEMIC”., Ciudad Autónoma de Buenos Aires, Argentina; [132]Dirección de Promoción y Prevención, Ministerio de Salud y Protección Social, Bogotá, Colombia; [133]The Research institute of Virology of the Republican specialized scientific practical medical center of epidemiology, microbiology, infections and parasitics diseases, Tashkent, Uzbekistan; [134]Gastroenterology, King Abdulaziz Medical City, Jeddah, Saudi Arabia; [135]Medizinische Klinik [2], St. Josefs-Hospital, Wiesbaden, Germany; [136]Medizinische Klinik 1, Goethe-University, Frankfurt am Main, Germany; [137]Department of Science and International Cooperation, Kazakh Science Center of Dermatology and Infectious Diseases, Almaty, Kazakhstan; [138]Department of Infectology and Travel Medicine, Medical Faculty, PJ Safárik University, Kosice, Slovakia; [139]Department of Infection and Immunity, Luxembourg Institute of Health, Esch sur Alzette, Luxembourg; [140]Hepatology and Gastroenterology Unit, Internal Medicine Department, Faculty of Medicine, Mansoura University, Mansoura, Egypt; [141]Egyptian Liver Research Institute and Hospital, Mansoura, Egypt, Mansoura, Egypt; [142]Communicable Disease Surveillance Centre, Health Protection, Public Health Wales, Cardiff, United Kingdom; [143]Gastroenterology and Hepatology, Port Said University, Port Said, Egypt and Egyptian Liver Research Institute and Hospital (ELRIAH), Mansoura, Egypt; [144]Division of Hepatology, Department of Medicine, Faculty of Health Sciences, University of Cape Town, Cape Town, South Africa; [145]Department of Internal Medicine, Division of Gastroenterology and Hepatology, Medical University of Graz, Graz, Austria; [146]Department of Medicine, University of Otago, Christchurch, Christchurch, New Zealand; [147]Gastroenterology, Christchurch Hospital, Christchurch, New Zealand; [148]Department of Hepatology & Gastroenterology, Charité Universitätsmedizin Berlin, Berlin, Germany; [149]Keck Medicine of University of Southern California, Los Angeles, United States; [150]Hepatology Department, Riga East University Hospital, Latvian Center of Infectious Diseases, Riga, Latvia; [151]Faculty of Medicine, University of Latvia, Riga, Latvia; [152]Internal Medicine & Infectious Diseases, University Medical Center Utrecht, Utrecht, Netherlands; [153]NUST School of Health Sciences, National University of Sciences and Technology (NUST), H-12, Islamabad, Pakistan; [154]Advanced Research Centre, European University of Lefke, Lefke, Northern Cyprus, TR-10 Mersin, Turkey; [155]Infection Control and Vaccines, Norwegian Institute of Public Health, Oslo, Norway; [156]Medical Data Analytics Centre (MDAC), Department of Medicine and Therapeutics, The Chinese University of Hong Kong, Hong Kong Special Administrative Region, China; [157]State Key Laboratory of Digestive Disease, Institute of Digestive Disease, The Chinese University of Hong Kong, Hong Kong Special Administrative Region, China; [158]Infectious Diseases, Sahlgrenska University Hospital, Gothenburg, Sweden; [159]Gastroenterology and hepatology, NROC, Astana, Kazakhstan; [160]Department of medicine III, RWTH University hospital Aachen, Aachen, Germany; [161]School of Medicine, College of Medicine and Center of Excellence for Metabolic Associated Fatty Liver Disease, National Sun Yat-sen University, Kaohsiung, Taiwan; [162]Hepatobiliary Section, Department of Internal Medicine, and Hepatitis Center, Kaohsiung Medical University Hospital, Kaohsiung Medical University, Kaohsiung, Taiwan; [163]School of Medicine and Hepatitis Research Center, College of Medicine, Kaohsiung Medical University, Kaohsiung, Taiwan; [164]Goethe-University, Department of Medicine, University Hospital, Frankfurt, Germany; [165]Liver Unit, Carmel Medical Center, Faculty of Medicine, Technion Institute of Technology, Haifa, Israel.

Journal of Hepatology, Volume 83

Supplemental information

Number of people treated for hepatitis C virus infection in 2014-2023 and applicable lessons for new HBV and HDV therapies

The Polaris Observatory Collaborators

The following authors in bold share first authorship: **Homie A Razavi, Imam Waked, Huma Qureshi, Loreta A Kondili, Ann-Sofi Duberg, Soo Aleman, Junko Tanaka, Jeffrey V Lazarus, and Daniel Low-Beer**.

Authors:

Homie A Razavi[1], Imam Waked[2], Huma Qureshi[3], Loreta A Kondili[4,5], Ann-Sofi Duberg[6], Soo Aleman[7], Junko Tanaka[8], Jeffrey V Lazarus[9,10], Daniel Low-Beer[11], Zaigham Abbas[12], Antoine Abou Rached[13], Alessio Aghemo[14,15], Inka Aho[16], Ulus S Akarca[17], Said A Al-Busafi[18], Waleed K Al-Hamoudi[19], Khalid Al-Naamani[20], Ahmed Sabry Alaama[21], Manahil M Aldar[22], Mohammed Alghamdi[23], Monica Alonso Gonzalez[24], Haleema Alserehi[25], Anil C Anand[26], Tarik Asselah[27], Abdullah M Assiri[28], Kostas Athanasakis[29], Rita Atugonza[30], Ziv Ben-Ari[31,32], Thomas Berg[33], Carlos E Brandão-Mello[34,35], Ashley S M Brown[36], Kimberly A Brown[37], Robert S Brown, Jr[38], Philip Bruggmann[39], Maurizia R Brunetto[40,41], Maria Buti[42], Hugo Cheinquer[43], Peer Brehm Christensen[44,45], Vladimir Chulanov[46,47], Laura E Cisneros Garza[48], Carla S Coffin[49], Nicola Coppola[50], Antonio Craxi[51], Javier Crespo[52,53], Fuqiang Cui[54], Olav Dalgard[55], Alethse De La Torre[56], Victor De Ledinghen[57], Douglas Dieterich[58], Sylvia Drazilova[59], Jean-François Dufour[60], Mohamed El-Kassas[61], Mohammed Elbadri[62], Gamal Esmat[63], Rafael Esteban Mur[64], Brandon Eurich[1], Diana Faini[65], Paulo R A Ferreira[66], Robert Flisiak[67], Sona Frankova[68], Giovanni B Gaeta[69], Ivane Gamkrelidze[1], Edward J Gane[70], Virginia Garcia[71], Javier García-Samaniego[72], Manik Gemilyan[73], Magnus Gottfredsson[74,75], Michael Gschwantler[76,77], Ana P M Gurski[78], Behzad Hajarizadeh[79], Saeed S Hamid[80], Angelos Hatzakis[81], Julian Hercun[82], Ivana Hockicková[83], Jee-Fu Huang[84], Bela Hunyady[85], Sharon J Hutchinson[86,87], Naoko Ishikawa[11], Kiyohiko Izumi[88], Antonio Izzi[89], Martin Janicko[59], Peter Jarcuska[59], Agita Jeruma[90,91], Asgeir Johannessen[92], Kulpash S Kaliaskarova[93], Jia-Horng Kao[94], Knut B Kielland[95], Nicolas Kodjoh[96], Shyamasundaran Kottilil[97], Pavol Kristian[83], Paul Y Kwo[98], Martin Lagging[99,100], Hilton Lam[101], Pablo Lázaro[102], Mei-Hsuan Lee[103], Sabela Lens[104], Valentina Liakina[105,106], Young-Suk Lim[107], Michael Makara[108], Michael Manns[109], Casimir Mingiedi Manzengo[110], Sadik Memon[111], Maria Cássia Mendes-Correa[112], Vincenzo Messina[113], Håvard Midgard[114], Niamh Murphy[115], Erkin Musabaev[116], Marcelo C M Naveira[117], Helen Nde[1], Francesco Negro[118], Nirada Nim[119], Ponsiano Ocama[120], Sigurdur Olafsson[121], Casimir E Omuemu[122], Javier Pamplona[123], Calvin Q Pan[124], George V Papatheodoridis[125], Nikolay Pimenov[46,126], Hossein Poustchi[127], Maria Giovanna Quaranta[128], Alnoor Ramji[129], Henna Rautiainen[130], Devin M Razavi-Shearer[1], Kathryn Razavi-Shearer[1], Ezequiel Ridruejo[131], Cielo Y Ríos-Hincapié[132], Shakhlo Sadirova[133], Faisal M Sanai[134], Christoph Sarrazin[135,136], Gulya Sarybayeva[137], Ivan Schréter[138], Carole Seguin-Devaux[139], Leandro S Sereno[24], Gamal Shiha[140,141], Josie Smith[142], Riham Soliman[143], Mark W Sonderup[144], C Wendy Spearman[144], Rudolf E Stauber[145], Catherine A M Stedman[146,147], Vana Sypsa[81], Frank Tacke[148], Norah A Terrault[149], Ieva Tolmane[150,151], Berend Van Welzen[152], Alexis S Voeller[1], Yasir Waheed[153,154], Carolyn Wallace[1], Robert N Whittaker[155], Vincent W-S Wong[156,157], Magdalena Ydreborg[158], Kakharman Yesmembetov[159,160], Ming-Lung Yu[161,162,163], Stefan Zeuzem[164], Eli Zuckerman[165].

[1]Center for Disease Analysis Foundation, Lafayette, United States; [2]Hepatology, National Liver Institute, Shebeen El Kom, Egypt; [3]Gastroenterologist, Focal Point Hepatitis, Ministry of National Health Services, Regulations and Coordination, Islamabad, Pakistan; [4]National Center for Global Health, Istituto Superiore di Sanita, Rome, Italy; [5]UniCamillus-Saint Camillus International University of Health Sciences, Rome, Italy; [6]Department of Infectious Diseases, Faculty of Medicine and Health, Örebro University, Örebro, Sweden; [7]Department of Infectious Diseases, Karolinska University Hospital, Stockholm, Sweden; [8]Epidemiology, Infectious Disease Control and Prevention, Hiroshima University, Hiroshima, Japan; [9]CUNY Graduate School of Public Health and Health Policy, New York City, United States; [10]Barcelona Institute for Global Health (ISGlobal), Hospital Clínic, University of Barcelona, Barcelona, Spain; [11]Global Hepatitis Programme, World Health Organization, Geneva, Switzerland; [12]Hepatogastroenterology, Dr. Ziauddin University Hospital, Karachi, Pakistan; [13]Internal Medicine - Gastroenterology, Faculty of Medical Sciences, Lebanese University, Beirut, Lebanon; [14]Department of Biomedical Sciences, Humanitas University, Pieve Emanuele, Italy; [15]Department of Gastroenterology, Humanitas Research Hospital IRCCS, Rozzano, Italy; [16]Infectious Diseases, Helsinki University Hospital, Helsinki, Finland; [17]Department of Gastroenterology, Ege University, Medical School, Izmir, Turkey; [18]Medicine, College of Medicine and Health Sciences, Sultan Qaboos University, Al-Khoudh, Oman; [19]Department of Medicine, King Saud University, Riyadh, Saudi Arabia; [20]Internal Medicine, Division of

JHEP

CTAT methods table

Tables for a "Complete, Transparent, Accurate and Timely account" (CTAT) are now mandatory for all revised submissions. The aim is to enhance the reproducibility of methods.

- Only include the parts relevant to your study
- Refer to the CTAT in the main text as 'Supplementary CTAT Table'
- Do not add subheadings
- Add as many rows as needed to include all information
- Only include one item per row

If the CTAT form is not relevant to your study, please outline the reasons why:

Register-based case-control study.

1.1 Antibodies

Name	Citation	Supplier	Cat no.	Clone no.

1.2 Cell lines

Name	Citation	Supplier	Cat no.	Passage no.	Authentication test method

1.3 Organisms

Name	Citation	Supplier	Strain	Sex	Age	Overall n number

1.4 Sequence based reagents

Name	Sequence	Supplier

1.5 Biological samples

Description	Source	Identifier

1.6 Deposited data

Name of repository	Identifier	Link

1.7 Software

Software name	Manufacturer	Version
Crystal Ball	Oracle	11.1.3708.0

1.8 Other (*e.g.* drugs, proteins, vectors etc.)

1.9 Please provide the details of the corresponding methods author for the manuscript:

Homie Razavi, hrazavi@cdafound.org, +1-314-8054884

2.0 Please confirm for randomised controlled trials all versions of the clinical protocol are included in the submission. These will be published online as supplementary information.
